# Metal-organic frameworks with linear and branched polyol backbones for dye removal

**DOI:** 10.1038/s41598-026-37325-0

**Published:** 2026-05-28

**Authors:** Safoora Gazvineh, Mohsen Adeli, Mohammad Nemati

**Affiliations:** Department of Organic Chemistry, Freie Universität Berlin, Lorestan University, Khorramabad, 68141-54316 Iran

**Keywords:** Metal-organic framework, Polyvinyl alcohol, Hyperbranched polymer poly(glycerol), MIL-101 (Fe), Dye removal, Chemistry, Environmental sciences, Materials science

## Abstract

In this study, novel metal-organic frameworks (MOFs) were synthesized using functionalized polyols as organic ligands. Polyvinyl alcohol (PVA) and hyperbranched polyglycerol (hPG) were first mesylated and subsequently modified with 5-aminoisophthalic acid, followed by coordination with iron (III) chloride hexahydrate to form PVA-MOF and hPG-MOF structures. Comprehensive characterization, including FTIR, NMR, XRD, SEM/EDX, BET, zeta potential, and UV–Vis DRS analyses, confirmed successful polymer functionalization, preserved MOF crystallinity, porous morphology, high surface area, elemental composition, optical properties, and surface charge relevant to dye adsorption. The resulting MOFs exhibited high adsorption capacities toward both cationic (Rhodamine B and Methylene Blue) and anionic (Fluorescein) dyes from aqueous solutions. Isotherm analysis using Langmuir and Freundlich models revealed that dye uptake was best described by the Langmuir isotherm, indicating monolayer adsorption on a homogeneous surface, with maximum adsorption capacities ranging from 128.17 to 135.34 mg g^−1^ depending on the dye and MOF type. Thermodynamic studies demonstrated that the adsorption process was endothermic, with increased entropy, and spontaneous for the anionic dye, while non-spontaneous for the cationic dyes. The materials also showed excellent structural stability and reusability potential over three adsorption-desorption cycles. The adsorption performance was further validated using real water samples, confirming that PVA-MOF and hPG-MOF are promising and reusable adsorbents for efficient dye removal in wastewater treatment.

**Supplementary Information:**

The online version contains supplementary material available at 10.1038/s41598-026-37325-0.

## Introduction

The rapid technological advancements of recent decades have raised significant environmental concerns, particularly regarding water pollution^1^. As water is an essential resource for life, protecting aquatic environments from contamination caused by population growth, urbanization, and industrialization has become a global priority^2^. In recent years, surface waters and waste streams have shown increased levels of persistent organic pollutants, especially synthetic dyes released by textile, paper, food, and cosmetic industries, which adversely affect aquatic life and human health due to their toxicity, carcinogenicity, and resistance to biodegradation^3–5^.

Various wastewater treatment technologies, including coagulation/flocculation, ion exchange, membrane separation, and adsorption, have been developed to remove such organic pollutants from water^6^. Among these, adsorption is an effective and economically viable technique due to its simplicity, high removal efficiency, and adaptability to various contaminant classes^7,8^.

Metal–organic frameworks (MOFs) are an emerging class of crystalline porous materials constructed from metal ions or clusters coordinated with organic ligands, exhibiting exceptionally high surface areas and tunable pore structures^9,10^. These unique properties have led to extensive research on MOFs in catalysis, gas separation, drug delivery, sensing, and environmental remediation, particularly the adsorption and catalytic removal of organic pollutants from aqueous environments^11–14^. Several comprehensive reviews have discussed the design, synthesis strategies, and removal mechanisms of MOFs and their composites in water purification applications^15–17^.

To further enhance MOF stability, processability, and reusability, polymer–MOF composites have been increasingly investigated^18^. The incorporation of polymers into MOF structures can improve mechanical strength and water stability while maintaining porosity. Polyvinyl alcohol (PVA) is a hydrophilic synthetic polymer with abundant hydroxyl groups that facilitate strong interactions with adsorbates, making it suitable for water treatment applications^19–21^. In contrast, hyperbranched polyglycerols (hPGs) offer a high density of functional groups, water solubility, and tunable architectures that are advantageous in adsorption-based remediation^22–24^.

. Despite extensive research on MOF-based adsorbents, the development of polyol-functionalized polymer–MOF composites remains relatively underexplored, particularly using functionalized polyols such as PVA and hPG for the simultaneous removal of both cationic and anionic dyes. This work addresses this gap by designing novel PVA-MOF and hPG-MOF composites with enhanced adsorption capacity, structural stability, and regeneration potential, demonstrating performance in real water samples.

In this study, PVA-MOF and hPG-MOF composites were synthesized and characterized using FTIR, SEM, EDX, XRD, BET, zeta potential measurements, UV-Vis DRS, and NMR spectroscopy. The elemental composition of the materials was evaluated using EDX analysis, which is consistent with CHNS data provided in the Supplementary Information. Their adsorption performance toward cationic dyes (Rhodamine B and Methylene Blue) and an anionic dye (Fluorescein) was evaluated under varying conditions of pH, temperature, and initial dye concentration. Adsorption isotherm models and thermodynamic analyses were applied to elucidate the adsorption mechanism, indicating the potential of these materials for efficient dye removal from contaminated water. Notably, the materials exhibited excellent structural stability and reusability, which are crucial for practical wastewater treatment applications.

## Materials and methods

### Materials

N, N-Dimethylformamide (DMF), ethanol, acetonitrile, acetone, chloroform, polyvinyl alcohol (PVA, Mw = 72,000 g mol^−1^), and hyperbranched poly(glycerol) (hPG, Mw = 5,000 g mol^−1^) were purchased from Sigma-Aldrich. 5-Amino isophthalic acid (AIP, 95%), hydrochloric acid (HCl, 37%), sodium hydroxide (NaOH, 98%), iron(III) chloride hexahydrate (FeCl₃·6 H₂O, 99%), Methylene Blue (C₁₆H₁₈ClN₃S, 99%), Rhodamine B (C₂₈H₃₁ClN₂O₃, 99%), and Fluorescein (C₂₀H₁₂O₅, 99%) were obtained from Merck. Triethylamine (Et₃N), methanesulfonyl chloride (MsCl), and potassium carbonate (K₂CO₃) were purchased from Sigma-Aldrich. All reagents were used without further purification. Dialysis bags (MWCO: 2 kDa and 14 kDa) were provided by BioTechCell.

### Experimental/synthesis

PVA and hPG were first mesylated to generate PVA-OMs and hPG-OMs. The mesylated polymers were subsequently conjugated with 5-amino isophthalic acid (AIP) to form PVA-AIP and hPG-AIP. Finally, these functionalized polymers were reacted with FeCl_3_.6H_2_O to form PVA-MOF and hPG-MOF. The resulting MOFs were purified by washing and drying. Detailed reaction conditions, purification steps, and yields are provided in the Supplementary Information.

### Characterization

The synthesized MOFs were characterized using the following techniques:

FTIR (400–4000 cm^−1^, KBr pellet) to confirm functionalization and coordination.

XRD to analyze crystallinity.

SEM/EDX for morphology and elemental composition.

BET surface area to determine porosity.

It should be noted that mechanical properties such as tensile strength or burst index are generally evaluated for self-supporting films or bulk materials. Since the synthesized MOFs and polymer–MOF composites in this study were obtained in powdered form, such mechanical measurements are not applicable.

Zeta potential to measure surface charge.

UV–Vis DRS and NMR spectroscopy to confirm chemical structure.

CHNS analysis was performed (results in Table S1).

### Adsorption experiments

Adsorption performance was tested for Rhodamine B, Methylene Blue, and Fluorescein. Stock solutions (100 mg L^−1^) were diluted to required concentrations. 3 mg of each MOF was added to 5 mL of dye solutions at 5, 10, and 20 ppm, and incubated for 24 h at room temperature without agitation. Dye concentrations after adsorption were measured using UV–Vis spectroscopy. Adsorption capacity (q_t_) and removal efficiency (%R) were calculated. The MOFs showed efficient removal of both cationic (Rh B, MB) and anionic (FL) dyes.The dye adsorption capacity (q_t_, mg g^−1^) and removal efficiency (%R) were calculated using the following equations:

1 $${q_t}=\left( {{C_0} - {\text{ }}{C_t}} \right) * V/m$$

2 $$\% R=\left( {{C_0} - {C_t}} \right)/C0 \times 100$$

where C_0_ and C_t_ are the initial and time-dependent dye concentrations (mg L^−1^), V is the volume of the solution (L), and m is the mass of the adsorbent (g)^25^.

## Results

**Fig. 1:**
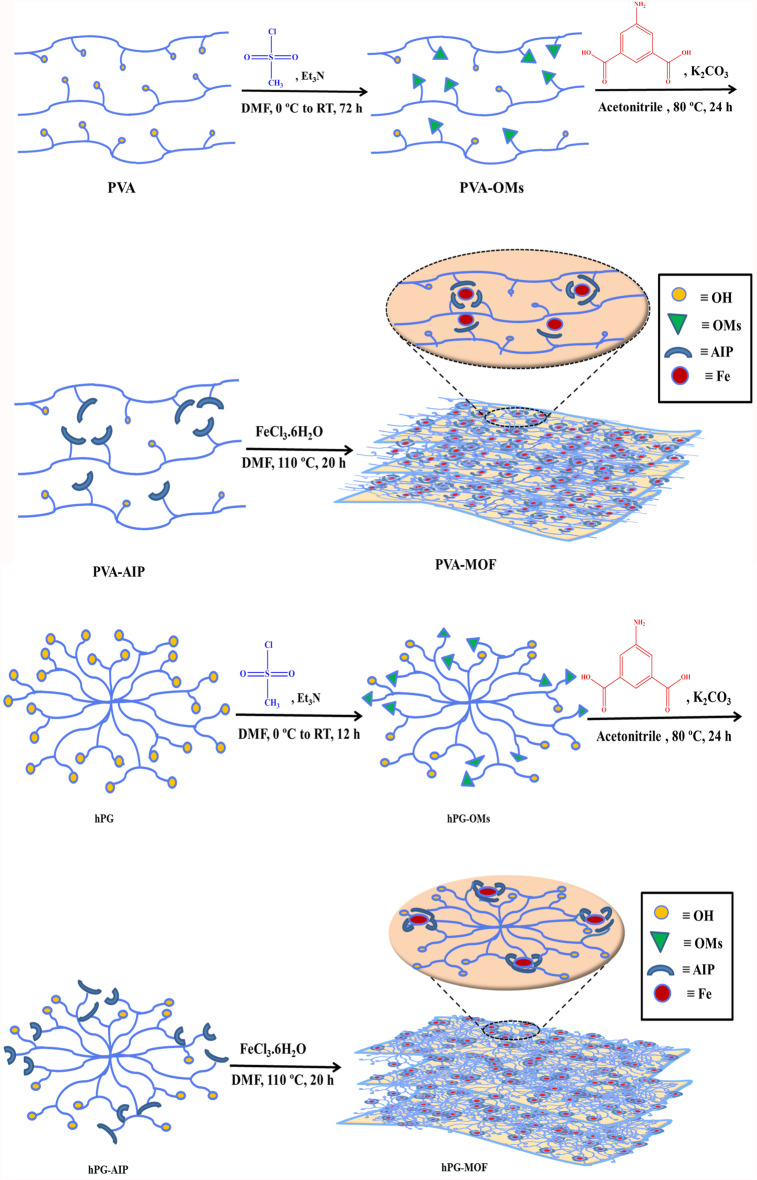
Schematic illustration of the synthesis of PVA-MOF and hPG-MOF. Following mesylation of PVA and hPG, the polymers were functionalized with 5-amino isophthalic acid (AIP) and subsequently subjected to a ligand exchange reaction with iron (III) chloride to yield the corresponding MOFs.

Metal–organic frameworks (MOFs) with polymeric backbones were synthesized by first conjugating ligands to the functional groups of polyols, followed by a ligand exchange reaction with iron (III) chloride (Fig. 1). Using this approach, MOFs with polyvinyl alcohol (PVA) and hyperbranched polyglycerol (hPG) matrices were successfully synthesized and by various spectroscopy and microscopy methods along with thermal and elemental analyses characterized.

**Fig. 2:**
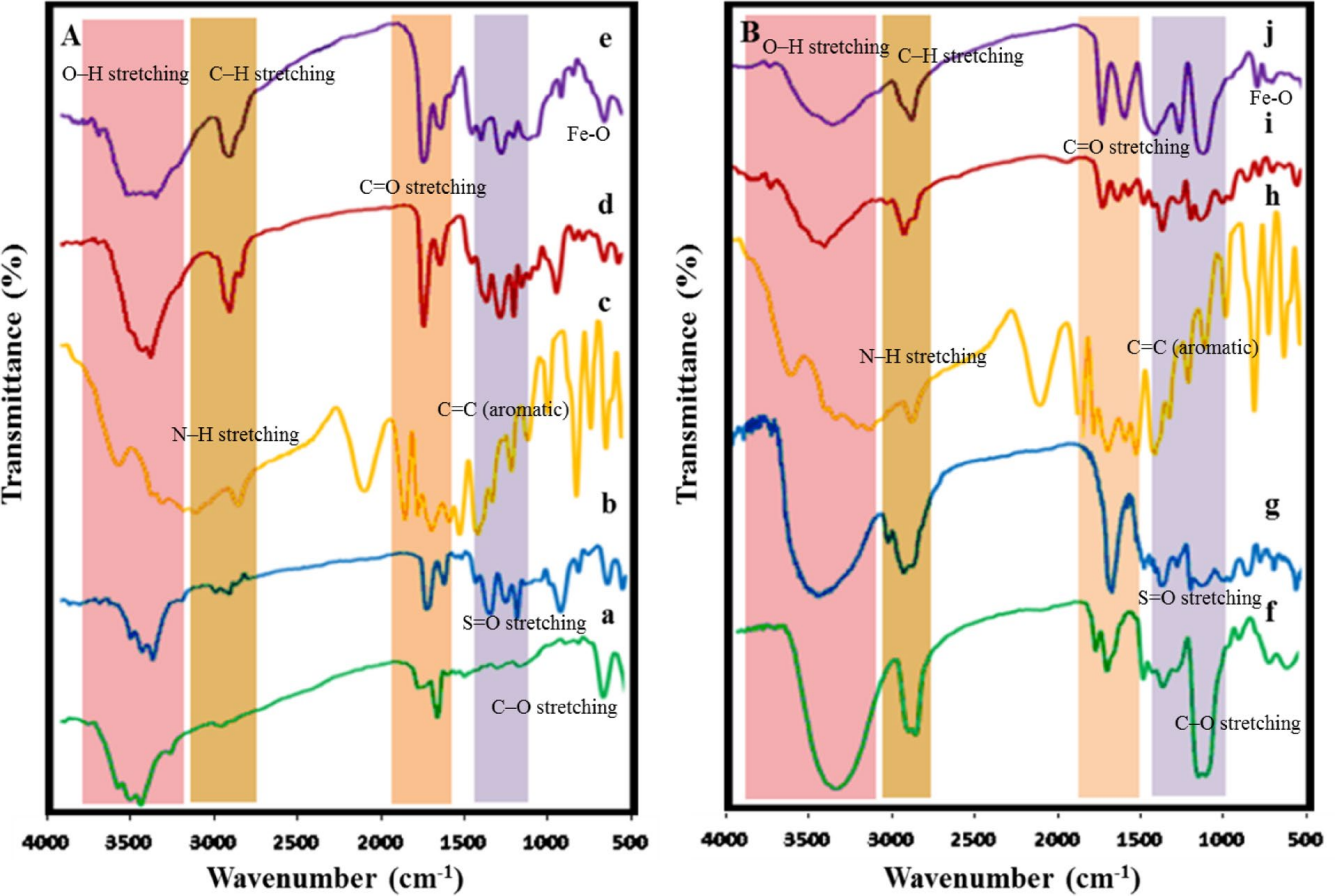
IR spectra of PVA-MOF (**A**) (a) PVA, (b) PVA-OMs, (c) AIP, (d) PVA-AIP, (e) PVA-MOF and hPG-MOF (**B**): (f) hPG, (g) hPG-OMs, (h) AIP, (i) hPG-AIP, (j) hPG-MOF and their precursors. The characteristic absorption bands corresponding to major functional groups are indicated in the spectra.

In the IR spectrum of PVA-OMs, the intensity of the O–H absorbance band characteristic of PVA is notably reduced, indicating substitution by mesylate groups (Fig. 2A). Additionally, sharp and intense absorbance bands observed in the range of 1100–1400 cm^−1^ are attributed to the S = O stretching vibrations of the mesyl functional groups (Fig. 2Ab). The appearance of characteristic N–H stretching bands around 3300–3500 cm^−1^ in AIP and AIP-functionalized polymers further confirms the successful incorporation of the amine-containing ligand into both PVA and hPG backbones.

In the spectrum of polyvinyl alcohol functionalized with 5-amino isophthalic acid (PVA-AIP), a broad O–H stretching band between 3238.26 and 3741.65 cm^−1^ corresponding to the carboxyl groups of the conjugated ligands, confirmed the successful attachment of 5-amino isophthalic acid (AIP) to the PVA backbone. Further evidence of PVA-AIP formation was provided by absorbance bands at 1627.81 cm^−1^ and 1552.59 cm^−1^, corresponding to aromatic C = C stretching of the benzene ring, and a band at 1398.30 cm^−1^, assigned to C = N imine stretching vibration (Fig. 2Ad).

In the IR spectrum of PVA-MOF, the O–H absorbance band associated with acidic groups involved in coordinative bonding with Fe (III) became broadened and decreased in intensity, indicating coordination to the metal centers within the MOF structure. Furthermore, the absorbance band at 1728.10 cm^−1^, attributed to the C = O stretching of the carboxyl group in the ligand, was significantly reduced, supporting the occurrence of a ligand exchange reaction and coordination of carboxylate groups to iron (III) (Fig. 2Ae)^26^.

Similar spectral changes confirmed the successful functionalization of hPG and the formation of the corresponding MOF. A decrease in the intensity of hydroxyl absorbance bands around 3357.84 cm^−1^, along with the appearance of a broad absorbance band in the range of 3100–3741.65 cm^−1^, indicated mesylation and subsequent attachment of the AIP ligand to the hPG backbone. Furthermore, a reduction in the intensity of carboxyl-related bands, as well as a decrease in the C = O absorbance bands at 1714.60 cm^−1^ and 1575.73 cm^−1^, provided further evidence for the formation of hPG-MOF through coordination of the carboxylate groups to iron(III) (Fig. 2B(j))^27^.

The functionalization of the polymeric precursors and synthesis of multifunctional ligands with a polymeric backbone were confirmed by nuclear magnetic resonance (NMR) spectroscopy.

**Fig. 3:**
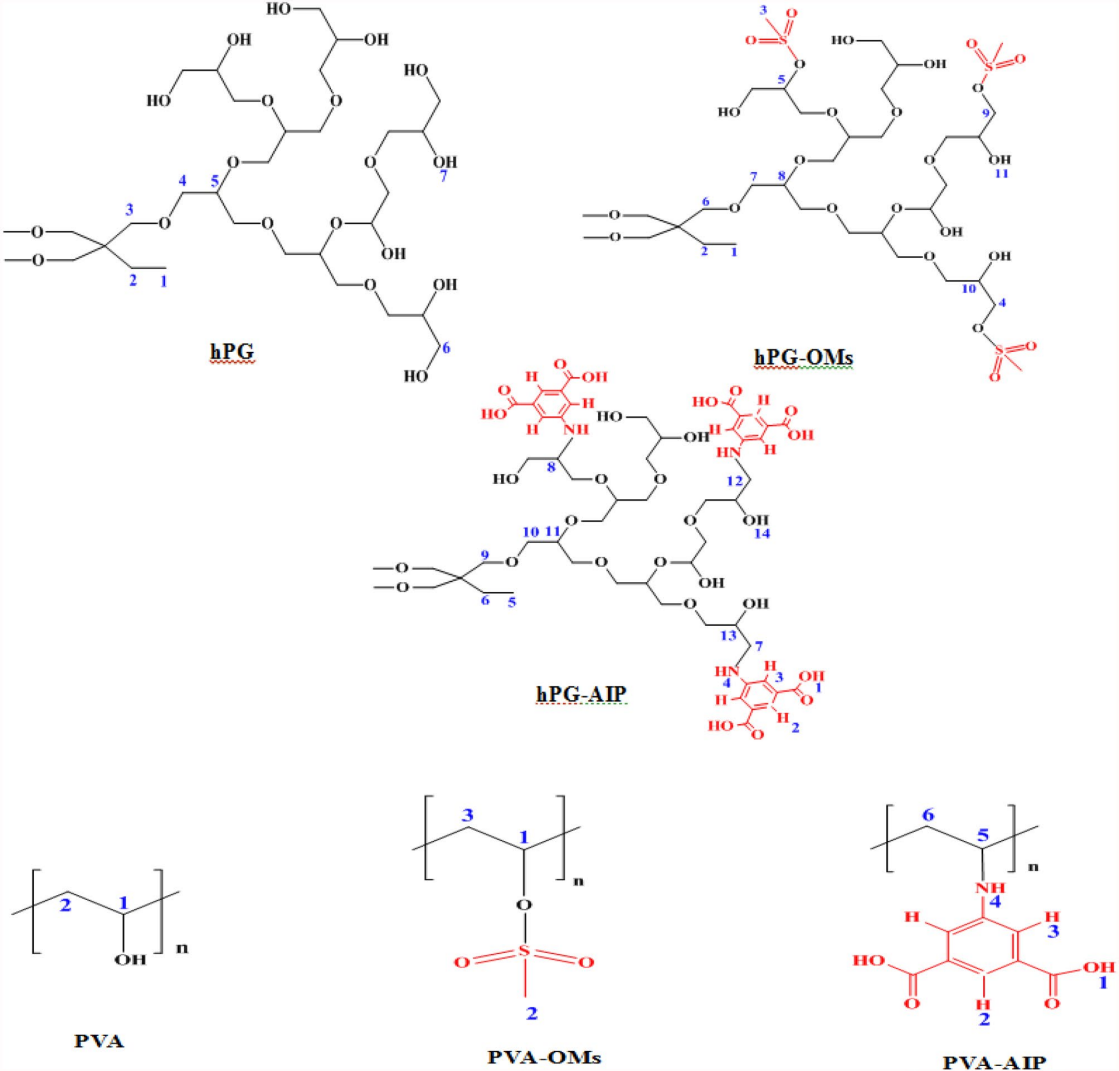
The successful functionalization of PVA and hPG was confirmed by ¹H and ^13^C NMR spectroscopy. The NMR spectra provided clear evidence for the conjugation of AIP ligands to the polymeric backbones, enabling the formation of multifunctional platforms suitable for MOF construction.

Following mesylation, the ¹H NMR spectra of PVA-OMs and hPG-OMs showed characteristic signals at δ = (2.3) and (2.1), respectively. Corresponding ^13^C NMR spectra displayed new signals at δ = (48.6) and (45.8), providing further evidence of successful mesylation of PVA and hPG (Fig. 3). Substitution of mesyl groups with 2-aminoterephthalic acid (AIP) resulted in the appearance of aromatic proton signals in the range of δ = 7.9–8.4 ppm in the ¹H NMR spectra of PVA-AIP and hPG-AIP, confirming successful conjugation of AIP ligands to the polymeric backbones. Additionally, new signals observed in the ^13^C NMR spectra within the range of δ = 120–163 ppm further support the incorporation of AIP ligands via mesyl group substitution (Fig. 3)^28,29^.

The crystallinity and long-range order of the synthesized MOFs were evaluated using X-ray diffraction (XRD). In the XRD pattern of pure PVA, a characteristic peak at 2θ = 19.5° corresponds to the (101) plane, while additional peaks at 2θ = 22.7° and 45.5° are assigned to the (200) and (111) planes, respectively. Following mesylation and subsequent conjugation of the ligand to PVA, the diffraction peaks became noticeably broader and weaker, indicating a significant increase in the amorphous character. This reduction in crystallinity suggests the formation of hydrogen bonding interactions between the PVA backbone and the amine and hydroxyl functional groups of the ligand ring.

**Fig. 4:**
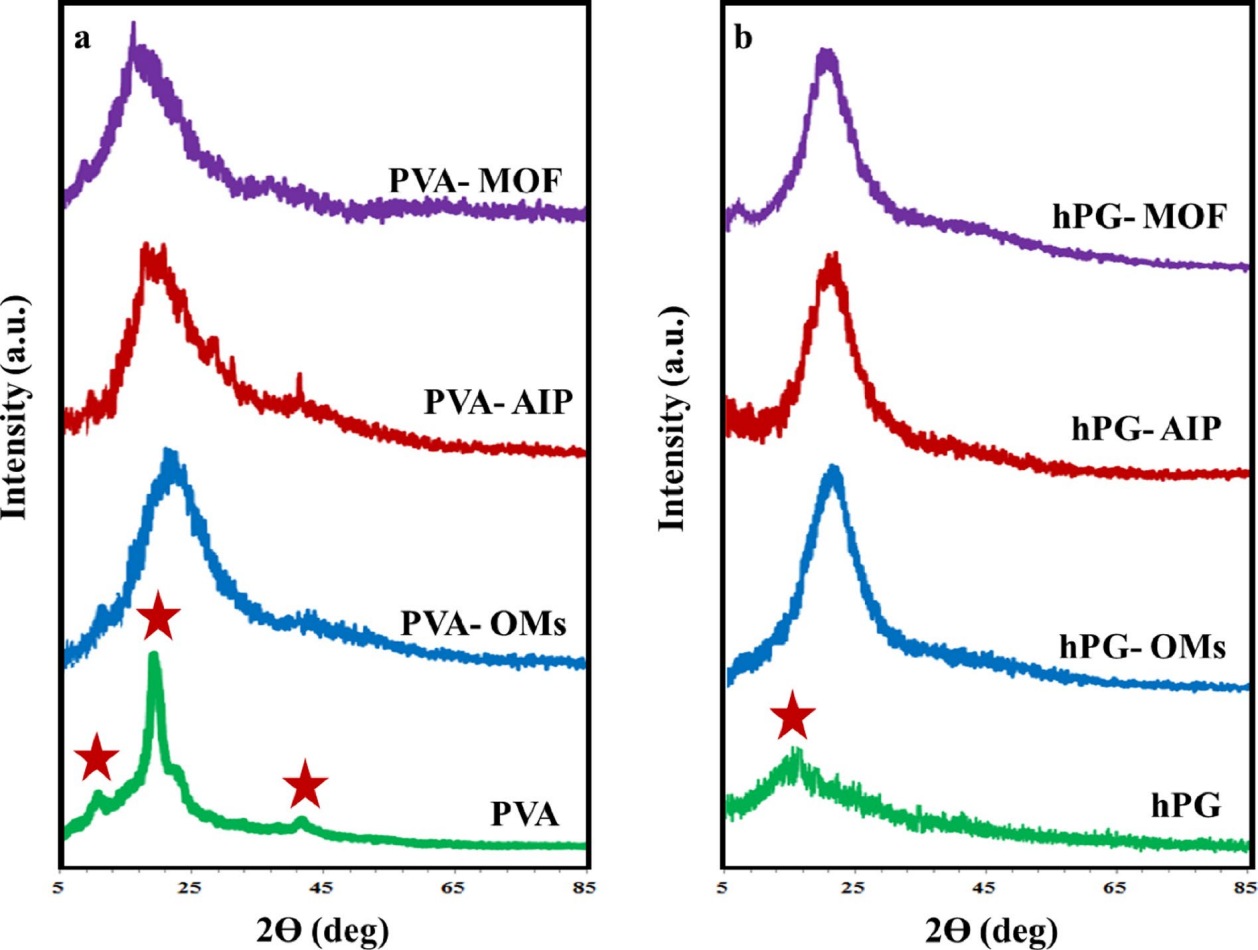
XRD diffractograms of PVA-MOF (**a**) and hPG-MOF (**b**) and their precursors.

The XRD pattern of PVA-MOF exhibited peaks at 2θ = 9.66° and 18.88°, similar to those observed in MIL-101, albeit with lower intensity, indicating successful incorporation of iron into the framework (Fig. 4a). As expected for amorphous materials, no distinct diffraction peaks were observed for hPG, hPG-OMs, or hPG-AIP. In contrast, the XRD pattern of hPG-MOF closely resembled that of PVA-MOF, also showing peaks at 2θ = 8.4° and 18.38° (Fig. 4b). These shared features with MIL-101 confirm the presence of iron-based coordination but with predominantly amorphous structures. The lack of sharp, well-defined peaks in both PVA-MOF and hPG-MOF is consistent with their polymeric backbones, which limit the formation of long-range crystalline order^30,31^.

Due to the predominantly amorphous nature of the polymer-based MOFs and the absence of sharp diffraction peaks, reliable determination of crystallite size and FWHM using the Scherrer equation was not applicable.

**Fig. 5:**
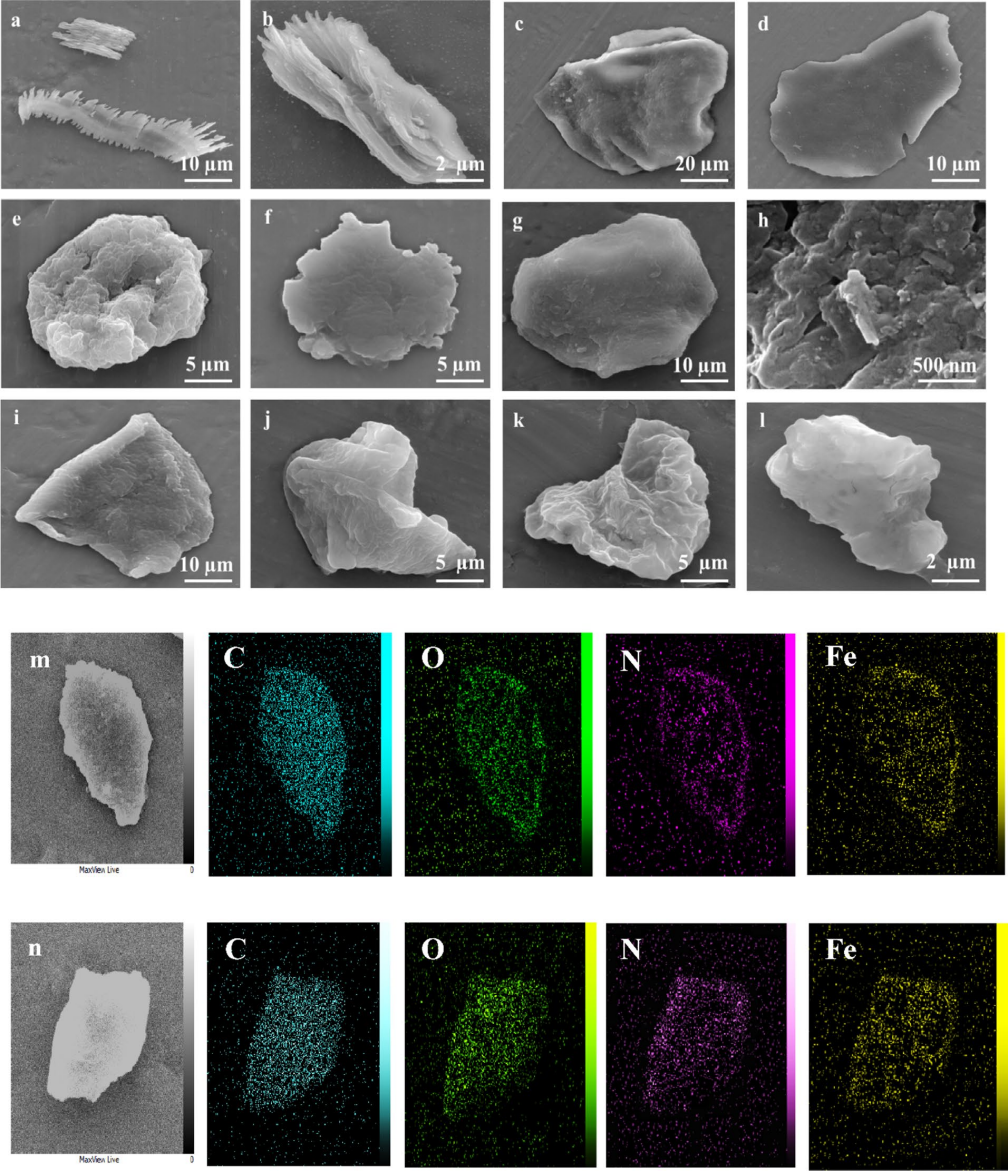
SEM images of PVA-MOF in DMF (**a**,**b**), deionized water (**c**,**d**) and chloroform (**e**,**f**). SEM images of hPG-MOF in DMF (**g**,**h**), deionized water (**i**,**j**), and chloroform (k, l). EDX elemental mapping and distribution for PVA-MOF (**m**) and hPG-MOF (**n**).

The morphology of PVA-MOF and hPG-MOF in three different solvents including DMF, deionized water, and CHCl_3_ was examined using scanning electron microscopy (SEM). Both materials exhibited sheet-like structures with lateral dimensions of several micrometers and well-defined edges. The observed solvent-dependent morphological variations suggest a high degree of responsivity of the polymeric backbones to the surrounding medium. Specifically, PVA-MOF retained a flat, sheet-like morphology in DMF, while displaying partially crumpled structures in deionized water and CHCl_3_. Conversely, hPG-MOF showed smooth, sheet-like surfaces in deionized water and CHCl_3_ but appeared crumpled in DMF. In all conditions, stacked sheets forming thick, layered frameworks were evident. These solvent-responsive morphological changes are attributed to the interplay between solvent polarity and the nature of the polymeric backbone, influencing the structural arrangement through specific solvent–polymer interactions (Fig. 5a–l)^32,33^.

The composition and elemental distribution of the materials were analyzed using energy-dispersive X-ray spectroscopy (EDX) (Fig. 5m, n). The spectra of PVA and hPG showed the presence of carbon and oxygen, consistent with their polymeric structures. In PVA-OMs and hPG-OMs, the appearance of sulfur peaks confirmed successful mesylation of the original polymers. Following functionalization with 5-amino isophthalic acid (AIP), nitrogen was detected in PVA-AIP and hPG-AIP, while sulfur content decreased, indicating successful conjugation of AIP to the polymer backbones. In PVA-MOF and hPG-MOF, the presence of iron, along with nitrogen, carbon, and oxygen, confirmed the formation of coordination bonds between the acidic functional groups of the ligands and Fe (III), consistent with MOF formation. Moreover, the composition of the synthesized materials was further confirmed by elemental analysis, and the results were consistent with the EDX data (Table S1).

Since iron originates exclusively from the MOF phase, the Fe weight% obtained from EDX analysis was used to qualitatively estimate the relative MOF content in the composites. Based on the higher Fe content observed for PVA-MOF compared to hPG-MOF, a higher MOF loading was inferred for PVA-MOF. Accordingly, the MOF content was estimated to be approximately 30–35 wt% for PVA-MOF and 20–25 wt% for hPG-MOF. These values represent approximate estimations rather than exact compositions, due to the semi-quantitative nature of EDX analysis.

Elemental analysis (CHNS) confirmed the composition of the synthesized materials, consistent with EDX results (Table S1).

**Fig. 6:**
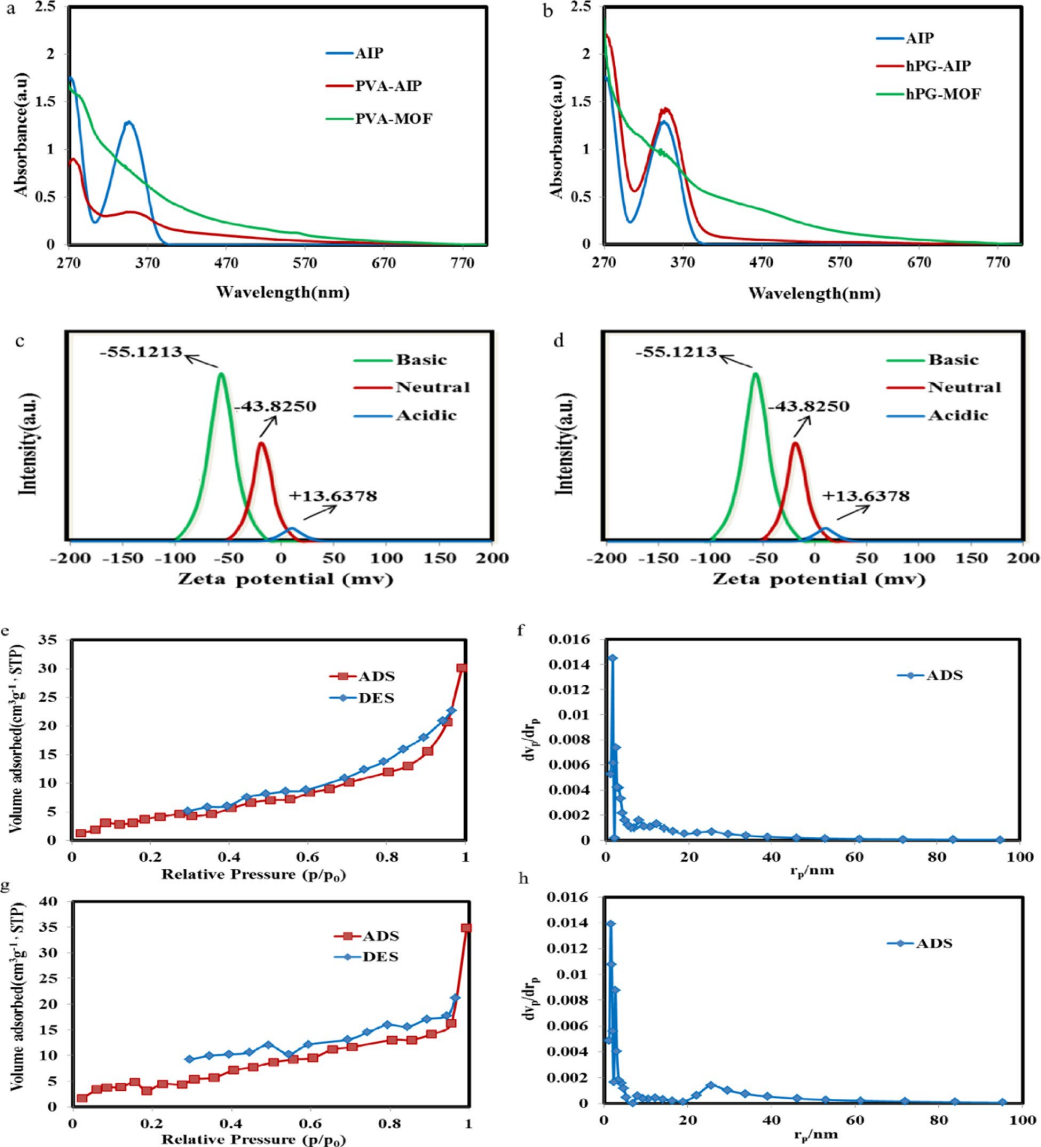
UV-Vis absorption spectra of PVA-MOF (**a**) and hPG (**b**) and the related precursors ligand. Zeta potential values of PVA-MOF (**c**) and hPG-MOF (**d**) under acidic, neutral and basic conditions. N_2_ adsorption-desorption isotherm (V vs. P/P_0_) of PVA-MOF (**e**) and hPG-MOF (**g**). Pore size distribution curve (dV/dRp vs. Rp) of PVA-MOF (**f)** and hPG-MOF (**h**).

The optical properties of the MOFs were investigated using UV-Vis spectroscopy. Both PVA-MOF and hPG-MOF exhibited broad absorption in the range of 270–670 nm, indicating a high density of metal–ligand interactions and the presence of aromatic rings within their structures (Fig. 6a and Table S1). This broad absorption is advantageous, as it suggests potential for photocatalytic applications such as reactive oxygen species (ROS) generation, although such activity is beyond the scope of the present study^34,35^.

The aim of this study was to evaluate the ability of the synthesized MOF frameworks to remove dye pollutants from aqueous solutions. To this end, the surface charge of the MOFs was investigated at various pH values, as it is a key factor influencing their adsorption performance. Due to the presence of carboxyl functional groups, the MOFs exhibited a highly negative surface charge under neutral and basic conditions, which shifted to a positive charge in acidic environments. This behavior is attributed to the pH-dependent protonation states of the functional groups: under basic and neutral conditions, carboxyl groups are deprotonated to carboxylates, conferring a negative charge, whereas in acidic conditions, carboxyl groups become protonated and amine groups are converted to their positively charged ammonium form (Fig. 6c,d)^36,37^.

**Fig. 7:**
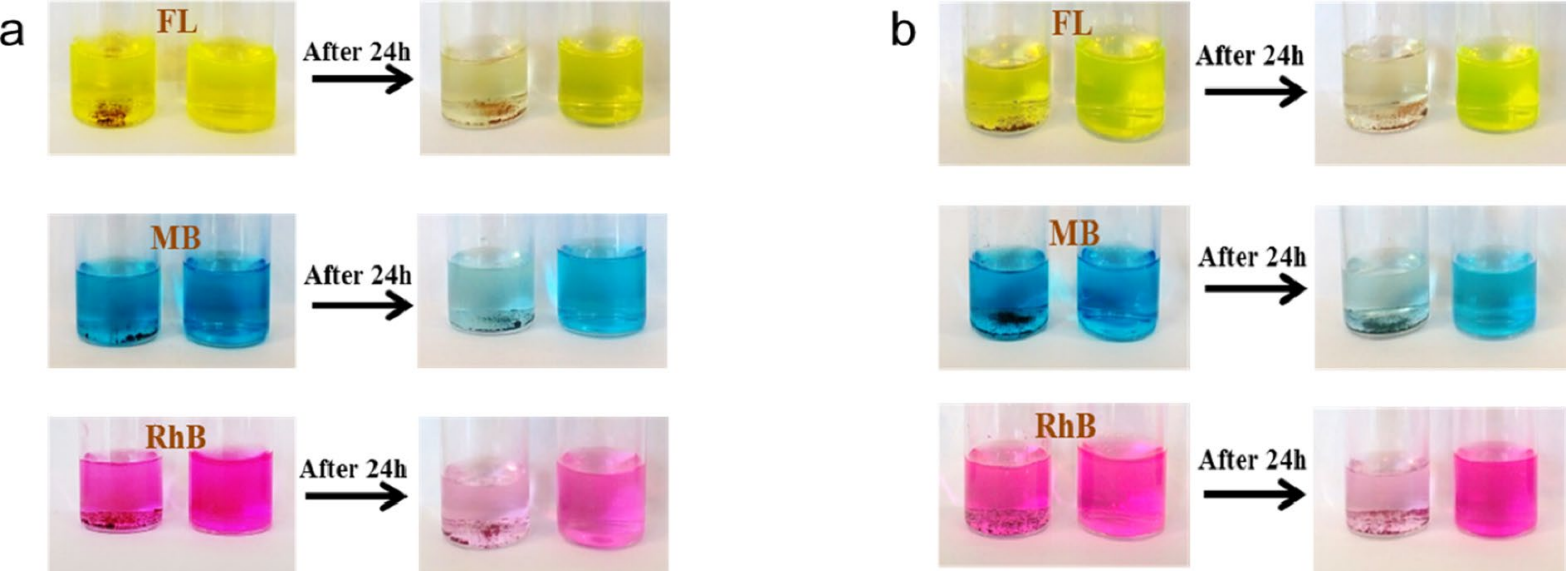
Digital photographs of PVA-MOF (**a**) and hPG-MOF (**b**) before and after incubation with different dye solutions (50 ppm, 5 mL). The MOFs were incubated with the dyes for 24 h at room temperature.

The porous structures of the synthesized PVA-MOF and hPG-MOF were characterized by nitrogen adsorption–desorption isotherms measured at 77 K and 81 kPa, corresponding to the saturation pressure of nitrogen at this temperature. The resulting isotherms are presented in Fig. 7 and summarized in Table S2. Both materials exhibited type IV isotherms, according to the IUPAC classification based on Brunauer et al., indicative of mesoporous structures with uniform pore distributions.

PVA-MOF and hPG-MOF showed total pore volumes of 0.048105 cm^3^ g^−1^ and 0.051623 cm^3^ g^−1^, respectively. The specific surface areas, calculated using the Brunauer–Emmett–Teller (BET) method, were approximately 15.509 m^2^ g^−1^ for PVA-MOF and 18.609 m^2^ g^−1^ for hPG-MOF. The Barrett–Joyner–Halenda (BJH) analysis revealed average pore diameters of 11.8 nm for PVA-MOF and 11.2 nm for hPG-MOF, with pore size distributions ranging from 2 to 50 nm (Fig. 7e-h). The measured specific surface areas were lower than expected, likely due to the collapse of the polymeric backbone in the dry state during nitrogen physisorption analysis. In the solution state, the polymeric network is expected to swell, maintaining a more open structure that would result in significantly higher accessible surface areas^38^.

## Adsorption studies

### Adsorption capacity

The discharge of organic dyes such as Methylene Blue (MB), Rhodamine B (RhB), and Fluorescein (FL) into water bodies poses significant environmental and health hazards due to their toxic and non-biodegradable nature. Therefore, developing efficient adsorbents for dye removal is of great importance^39^.

MOF–polymer composites combine the high surface area and porosity of MOFs with the chemical functionality and flexibility of polymers, resulting in materials with excellent adsorption capabilities for various dye molecules. In this study, 1 mg of the synthesized PVA-MOF or hPG-MOF was dispersed in 5 mL of dye solution (50 ppm) and subjected to sonication followed by stirring for 24 h at 25 °C. As shown in Fig. 7, both adsorbents exhibited high adsorption capacities toward all three tested dyes. These results highlight the strong potential of PVA-MOF and hPG-MOF composites for efficient dye removal in aqueous environments.

The maximum adsorption capacities of PVA-MOF for the cationic dyes rhodamine B (Rh B) and methylene blue (MB), as well as the anionic dye fluorescein (FL), were 128.17 mg g^−1^, 128.24 mg g^−1^, and 124.77 mg g^−1^, respectively. Under the same conditions, hPG-MOF exhibited adsorption capacities of 131.46 mg g^−1^ for Rh B, 135.34 mg g^−1^ for MB, and 128.31 mg g^−1^ for FL (Table S3). While PVA-MOF showed superior performance in the removal of the anionic dye, hPG-MOF demonstrated significantly higher adsorption capacities for cationic dyes compared to its linear counterpart.

Interestingly, both MOFs were able to adsorb the anionic FL dye effectively, despite their negatively charged surfaces under neutral and basic conditions. This observation suggests that electrostatic interactions are not the sole driving force governing dye adsorption; other mechanisms, such as π–π stacking, hydrogen bonding, and coordination interactions, may also play a significant role.

### Effect of pH

**Fig. 8:**
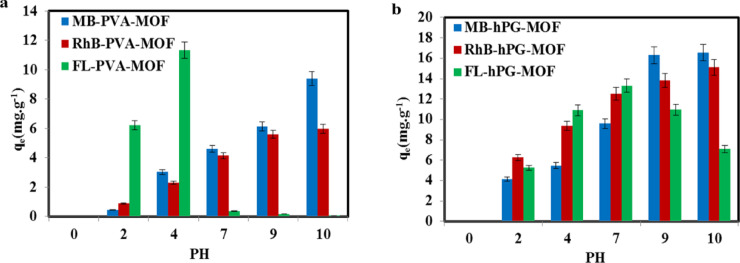
(**a**) Effect of pH on the adsorption capacity of PVA-MOF toward MB, RhB, and FL dyes, demonstrating the strong pH dependence of adsorption performance. (**b**) Effect of pH on the adsorption capacity of hPG-MOF toward MB, RhB, and FL, highlighting the enhanced adsorption efficiency and stability over a wide pH range compared with PVA-MOF.

The solution pH is a critical parameter influencing the adsorption behavior of dye molecules, as it governs both the surface charge of the adsorbent and the ionization state of the dyes. The effect of pH on the adsorption of Methylene Blue (MB), Rhodamine B (RhB), and Fluorescein (FL) was investigated over a pH range of 2–10 using PVA-MOF and hPG-MOF adsorbents (Fig. 8a,b). The initial pH of the dye solutions was approximately 7, and acidic or alkaline conditions were adjusted by adding 0.1 M HCl or 0.1 M NaOH, respectively.

As shown in Fig. 8a,b, the adsorption capacities of both PVA-MOF and hPG-MOF exhibited pronounced pH dependence. Under acidic conditions, the adsorption of cationic dyes (MB and RhB) decreased due to increased protonation of surface functional groups, leading to electrostatic repulsion. With increasing pH, enhanced adsorption of cationic dyes was observed, which can be attributed to the development of negatively charged surface sites, consistent with the zeta potential measurements.

In contrast, the adsorption of the anionic dye Fluorescein showed a different pH-dependent trend. Despite electrostatic repulsion at neutral and alkaline pH values, significant adsorption was still achieved, indicating that non-electrostatic interactions such as hydrogen bonding, π–π stacking, and coordination interactions contribute to dye uptake. Overall, hPG-MOF demonstrated superior adsorption performance and broader pH tolerance compared to PVA-MOF, highlighting the beneficial role of hyperbranched functionalization in stabilizing adsorption behavior across a wide pH range.

## Discussion

Table S3 presents a comparison of the adsorption capacities of the synthesized MOFs with those of analogous materials reported in the literature. As summarized in Table S3, the adsorption performance of PVA-MOF and hPG-MOF toward methylene blue is comparable to that of recently reported MOF-based adsorbents, including systems specifically developed for MB removal. Although some reported MOFs exhibit higher adsorption capacities, they often rely on fully aromatic frameworks or higher metal contents. In contrast, the polymer-based architectures employed in this study enable competitive dye removal efficiency while reducing metal loading and minimizing the potential risk of toxic ligand leaching, thereby enhancing environmental compatibility.

### Adsorption kinetic studies

Adsorption kinetic studies were performed to investigate the rate and mechanism of dye uptake by the synthesized PVA-MOF and hPG-MOF adsorbents. The experimental data were analyzed using both pseudo-first-order and pseudo-second-order kinetic models to determine the rate-controlling steps of the adsorption process.

The pseudo-first-order kinetic model is described by the following linear equation^40^. :

3 $$\ln \left( {{q_e} - {q_t}} \right)=\ln {\text{ }}{q_e} - {k_1}t$$

where q_e_ and q_t_ (mg g^−1^) represent the adsorption capacities at equilibrium and at time t (min), respectively, and k_1_ (min^−1^) is the pseudo-first-order rate constant. Linear plots of ln(q_e_ - q_t_) versus time were used to determine the kinetic parameters and correlation coefficients (R^2^). The corresponding plots are provided in the Supplementary Information (Figure S2).

The pseudo-second-order kinetic model is expressed as:

4 $$t/{q_t}=1/\left( {{k_2}q_{e}^{2}} \right)+t/{q_e}$$

where k_2_ (g mg^−1^ min^−1^) is the pseudo-second-order rate constant. Plots of t/q_t_ versus time were employed to calculate q_e_ and k_2_ values. The kinetic parameters derived from both models are summarized in Table S4.

**Fig. 9:**
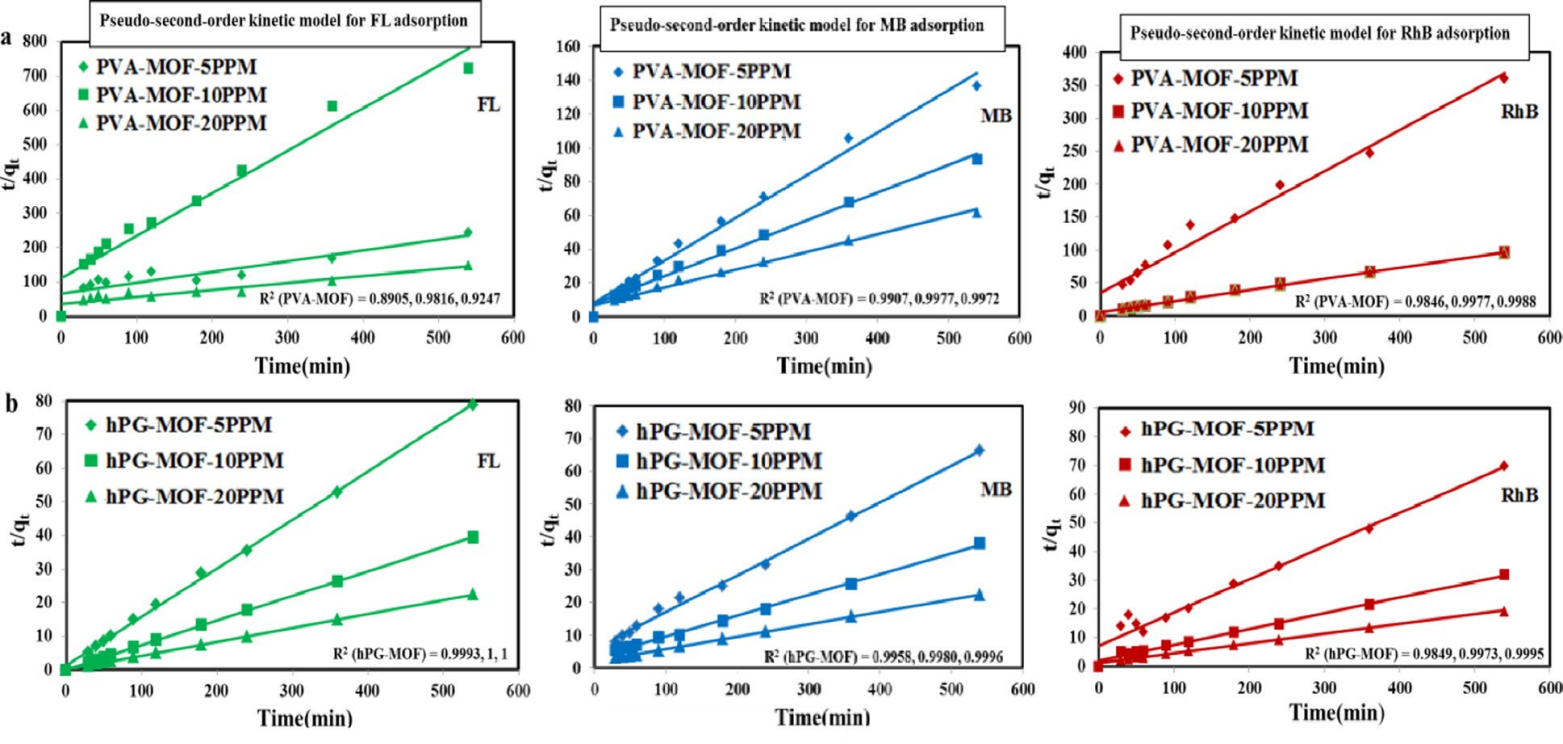
Pseudo-second-order kinetic model describing the adsorption of Methylene Blue (MB), Rhodamine B (Rh B), and Fluorescein (FL) onto PVA-MOF and hPG-MOF. The strong correlation between experimental data and the model suggests that chemisorption is the dominant mechanism governing dye uptake. All experiments were performed under constant shaking conditions to assess the adsorption kinetics and capacities of the composites for the selected dyes.

As shown in Fig. 9, the experimental adsorption data for Methylene Blue (MB), Rhodamine B (RhB), and Fluorescein (FL) exhibited a significantly better fit to the pseudo-second-order kinetic model, as evidenced by the higher R^2^ values displayed directly on the plots. This behavior suggests that the adsorption process is predominantly governed by chemisorption, involving valence forces through electron sharing or exchange between the dye molecules and the active sites of the polymer–MOF composites.

### Adsorption isotherm studies

Adsorption isotherm studies were carried out to evaluate the interaction between dye molecules and the synthesized PVA-MOF and hPG-MOF adsorbents under equilibrium conditions. The experimental adsorption data were analyzed using the Langmuir and Freundlich isotherm models to investigate the adsorption behavior and surface characteristics of the materials.

The corresponding isotherm plots are presented in Figure S3, and the calculated isotherm parameters are summarized in Tables S5 and S6. For all three dyes, namely Fluorescein (FL), Methylene Blue (MB), and Rhodamine B (RhB), the Langmuir model exhibited higher correlation coefficients (R^2^) compared to the Freundlich model, indicating that the adsorption process is better described by monolayer adsorption on a relatively homogeneous surface.

The maximum adsorption capacities (q_max_) predicted by the Langmuir model further confirm the high affinity of the polymer–MOF composites toward the investigated dyes. The favorable adsorption behavior can be attributed to the synergistic contribution of electrostatic interactions, π–π stacking, and hydrogen bonding between the dye molecules and the functional groups (e.g., hydroxyl, carboxyl, and amino groups) present on the polymeric MOF framework.

According to the Langmuir isotherm model, dye molecules are uniformly adsorbed within the cavities of the MOFs with polymeric matrices, indicating a homogeneous adsorption surface. This uniform adsorption behavior suggests that the PVA-MOF and hPG-MOF possess a structurally consistent and well-defined scaffold. The calculated maximum adsorption capacities for Fluorescein, Methylene Blue (MB), and Rhodamine B (Rh B), based on the Langmuir equation, are summarized in Table S5.

### Thermodynamic studies

**Fig. 10:**
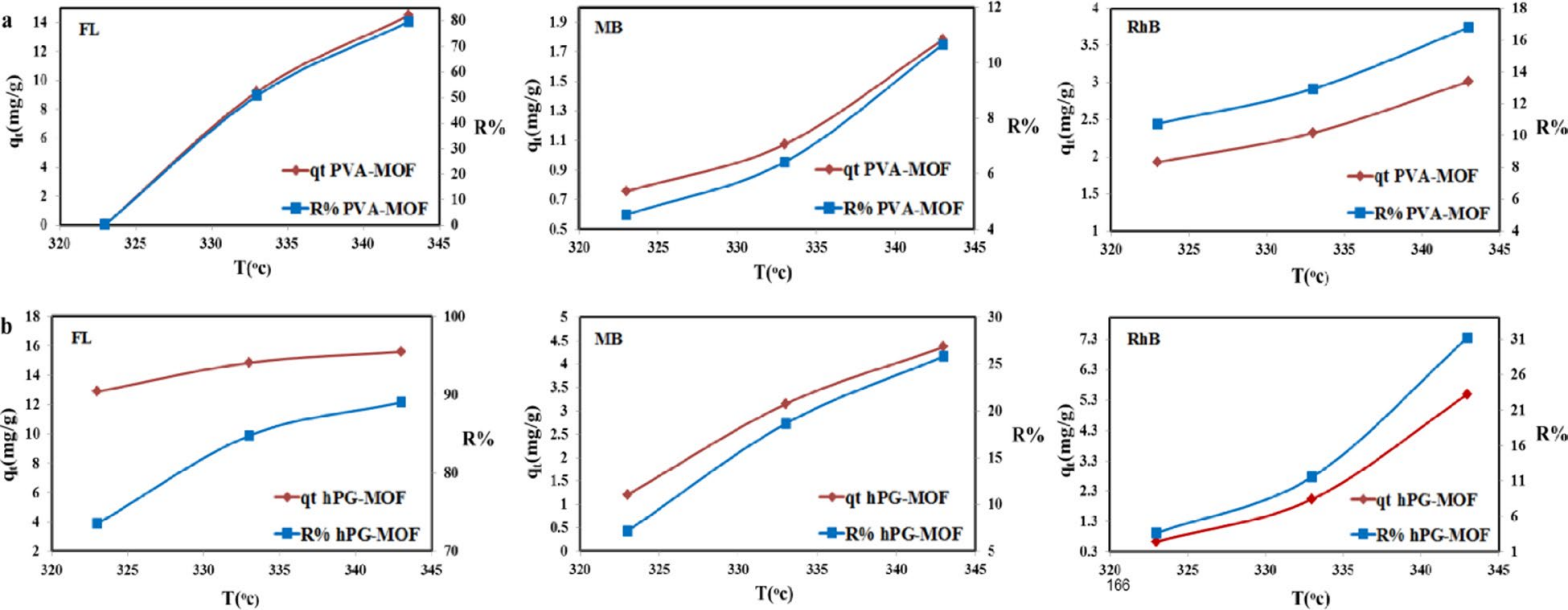
Percentage dye removal efficiency (%R) and adsorption capacity (q_t,_ mg g^−1^) of PVA-MOF (**a**) and hPG-MOF (**b**) for Methylene Blue (MB), Rhodamine B (Rh B), and Fluorescein (FL) at various temperatures. Dye solutions (10 mg L^−1^) were treated with each composite under shaking conditions, and dye removal was quantified using UV-Vis spectroscopy. Both composites demonstrated improved adsorption performance with increasing temperature, highlighting their potential for thermally responsive dye removal applications.

Thermodynamic studies were conducted to gain deeper insight into the feasibility and nature of the dye adsorption process on PVA-MOF and hPG-MOF adsorbents (Fig. 10, Tables S7, S8). The equilibrium adsorption data obtained at different temperatures were used to calculate the thermodynamic parameters, including Gibbs free energy change (ΔG°), enthalpy change (ΔH°), and entropy change (ΔS°).

The adsorption equilibrium constant (K_ad_) was determined from the ratio of equilibrium dye concentrations, and the thermodynamic parameters were calculated using the following relationships^40^:

5 $$Ln{\text{ }}{k_{ad}}=\Delta S/R - \Delta H/RT$$

6 $$\Delta {G^0}=\Delta {H^0} - T\Delta {S^0}$$

where K_ad_ is the adsorption equilibrium constant, C_0_ and C_e_ are the initial and equilibrium dye concentrations (mg L^−1^), respectively, R is the universal gas constant (0.008314 kJ mol^−1^ K^−1^), and T is the absolute temperature (°K).

The plots of lnK_ad_ versus 1/T are presented in Figure S4, and the calculated thermodynamic parameters (ΔG°, ΔH°, and ΔS°) are summarized in Table S8. The positive values of ΔH° indicate that the adsorption process is endothermic, suggesting that higher temperatures favor dye uptake. Additionally, the positive ΔS° values reflect an increase in randomness at the solid–liquid interface during adsorption, which can be attributed to desolvation effects and structural rearrangements of dye molecules upon interaction with the adsorbent surface.

The calculated ΔG° values reveal that the adsorption of the anionic dye (Fluorescein) is spontaneous under the investigated conditions, whereas the adsorption of cationic dyes (Methylene Blue and Rhodamine B) is non-spontaneous, although thermodynamically more favorable at elevated temperatures. These results confirm that the adsorption process is governed by a combination of electrostatic interactions and hydrogen bonding between the dye molecules and the functional groups present on the polymer–MOF frameworks.

To further elucidate the adsorption process, the mechanism of dye uptake by PVA-MOF and hPG-MOF is proposed as follows.

### Adsorption mechanism

**Fig. 11:**
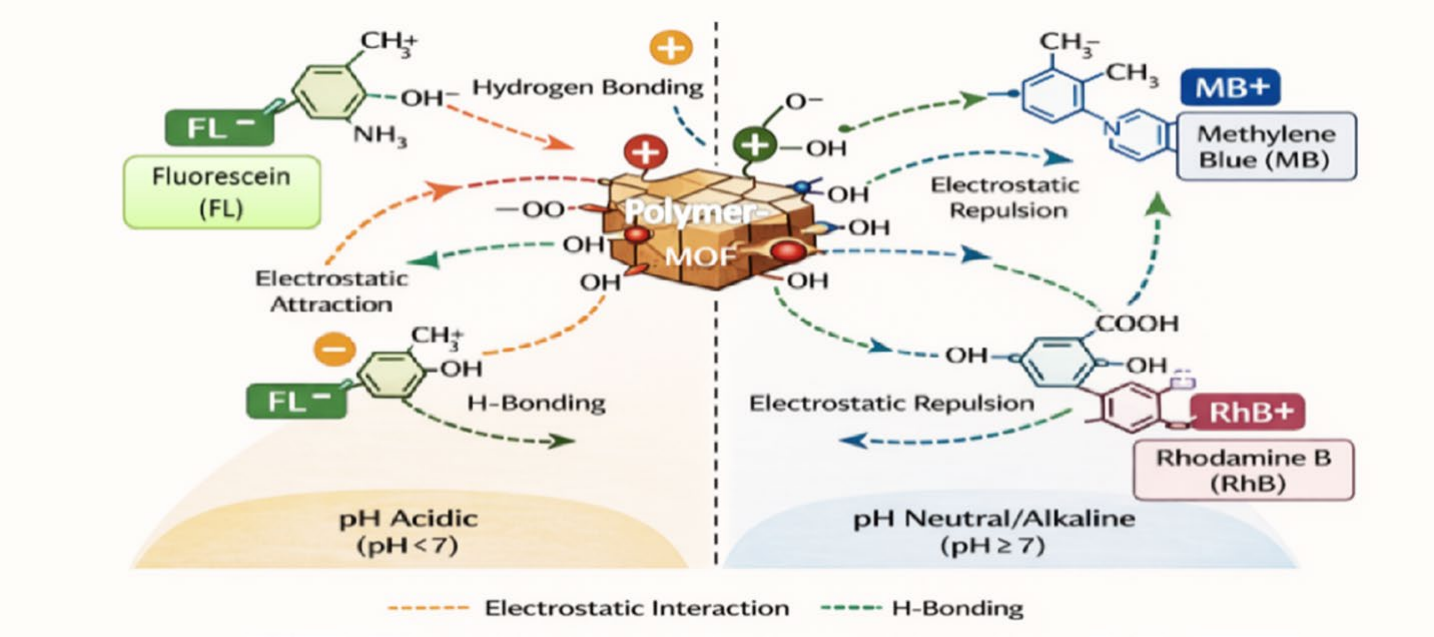
Proposed adsorption mechanism of cationic (MB, RhB) and anionic (FL) dyes onto PVA-MOF and hPG-MOF. The schematic illustrates hydrogen bonding and electrostatic interactions between functional groups of the polymer–MOF composites and dye molecules under different pH conditions.

The adsorption of cationic dyes (Rhodamine B and Methylene Blue) and anionic dye (Fluorescein) onto PVA-MOF and hPG-MOF is governed primarily by hydrogen bonding and electrostatic interactions. The abundant hydroxyl and carboxyl groups on the polymer–MOF composites facilitate strong hydrogen bonding with dye molecules, while the surface charge of the adsorbents, as indicated by zeta potential measurements, promotes electrostatic attraction or repulsion depending on the dye’s ionic nature. This combined effect leads to efficient adsorption of both cationic and anionic dyes onto the MOF surfaces, resulting in high removal efficiency. A schematic illustration summarizing these interactions is provided in Fig. 11.

### Real water samples

The findings indicated that both removal efficiency and adsorption capacity reach their maximum around pH 10 for Rh B and MB and decline at higher pH values. In basic mediums MB and Rh B are not positively charged, therefore electrostatic interaction with the negatively charged MOFs is not a driving force. On the other side FL was considerably adsorbed by hPG-MOF in basic medium while both dye and MOF are negatively charged. One possible explanation for the observed pH-dependent adsorption behavior is that, at higher pH values, the carboxyl groups of the AIP ligand become deprotonated, resulting in increased negative surface charge within the MOF pores. This electrostatic repulsion among negatively charged sites may lead to pore expansion or enhanced accessibility. As a result, the adsorption capacity increases, primarily through hydrogen bonding and π–π interactions rather than electrostatic attraction. Adsorption of FL by PVA-MOF was not significant in basic pH, indicating a major role for the electrostatic repulsion in the case.

Given that, in real environmental matrices such as municipal water, other elements coexist simultaneously, it is essential to evaluate the adsorption performance of the selected adsorbents under actual conditions. To this end, removal efficiency and adsorption capacity were assessed using samples of municipal water, Khoramrood River water, and Stone Stormwater, each containing dye concentrations of 50 ppm.

Samples of each water source were analyzed prior to treatment to determine their initial dye concentrations. Subsequently, 1 mg of the adsorbent was added to each solution, and the mixtures were stirred for a predetermined period. The residual dye concentrations were measured at specific time intervals to evaluate the adsorption kinetics. The removal efficiency and adsorption capacity were then calculated based on the initial and final dye concentrations. The results are summarized in tables S9. As shown in the results of the experiment with deionized water, both MOF adsorbents performed well in the adsorption of dyes. The adsorption capacity data showed distinct patterns for each dye: methylene blue showed the highest adsorption efficiency (128.24–135.34), followed by rhodamine B (128.17–131.46), while fluorescein showed the lowest adsorption capacity (124.77–128.31). The results showed that hPG-MOF consistently outperformed PVA-MOF in all types of dyes, with the most pronounced difference observed in the adsorption of methylene blue. These results were also confirmed in subsequent experiments with real waters (rock storm water, river water, and drinking water), indicating that both adsorbents also perform well in real environmental conditions.

### Reusability and stability

**Fig. 12:**
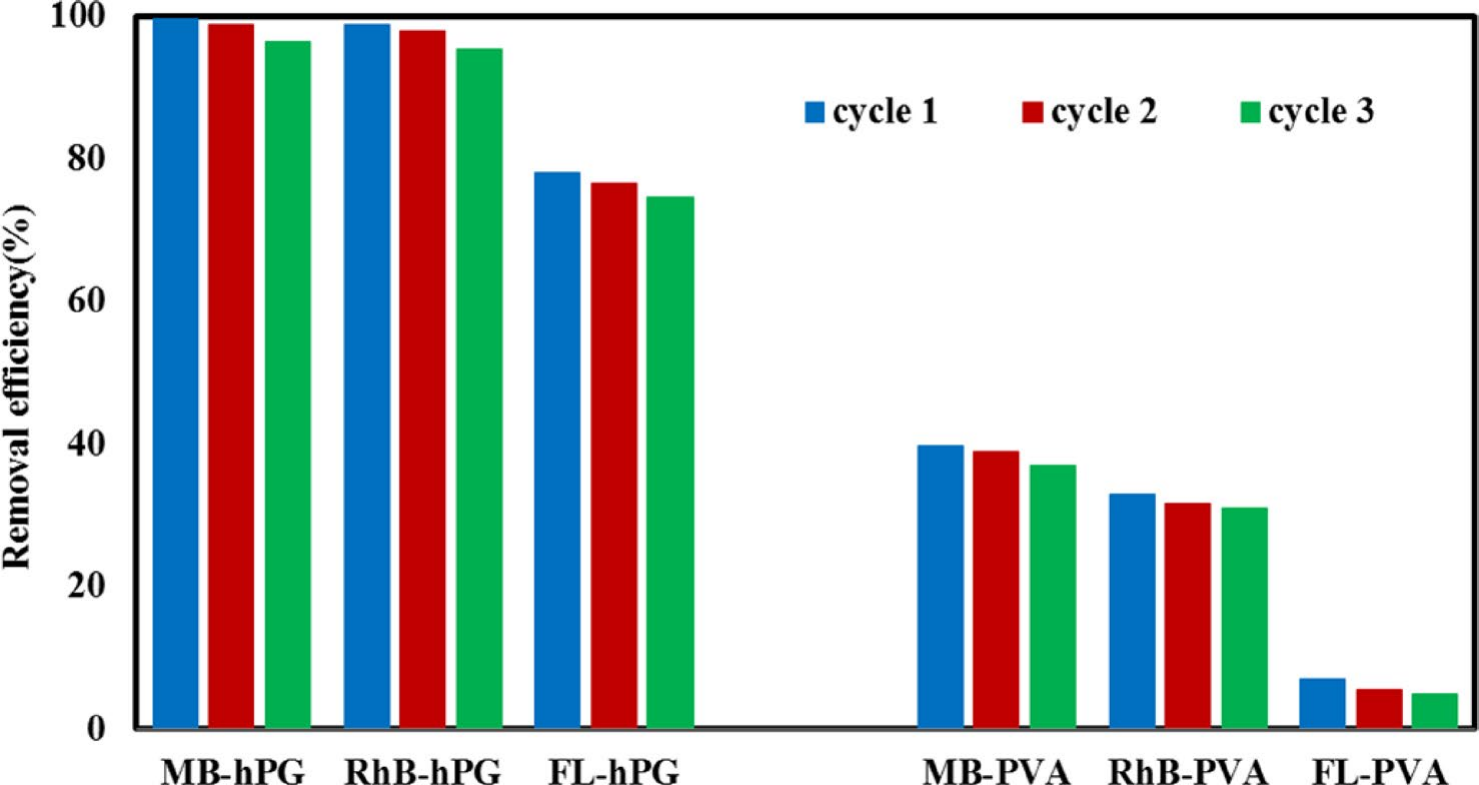
Reusability and regeneration performance of PVA-MOF and hPG-MOF adsorbents over three consecutive adsorption–desorption cycles for MB, RhB, and FL dyes, confirming the superior recyclability and cycle stability of hPG-MOF under repeated aqueous operation.

The stability and reusability of the adsorbents are essential for their practical application in dye removal from aqueous media. To assess these properties, 1 mg of each MOF was employed to adsorb dyes (10 mg L^− 1^) from aqueous solutions. In the first cycle, both PVA-MOF and hPG-MOF demonstrated near-complete dye removal. After each cycle, the adsorbents were regenerated by washing with acetone to desorb the dye molecules, followed by drying at 70 °C before reuse. As shown in Fig. 12, the hPG-MOF adsorbent exhibited remarkable stability over three consecutive adsorption–desorption cycles, demonstrating robust structural integrity and excellent reusability under repeated aqueous operation. Specifically, hPG-MOF consistently delivered high removal efficiencies for all tested dyes (MB, RhB, and FL), with MB showing 99.82%, 98.95%, and 78.21%; RhB showing 98.96%, 97.94%, and 76.69%; and FL showing 96.57%, 95.48%, and 74.75% across cycles 1–3, respectively.

In contrast, the PVA-MOF composite displayed a pronounced decline in removal efficiency upon successive regeneration cycles (MB: 39.69%, 32.99%, and 6.94%; RhB: 38.91%, 31.60%, and 5.60%; FL: 37.08%, 30.97%, and 4.88%), indicating partial loss of accessible adsorption sites and reduced framework robustness. This clear performance difference highlights the critical role of hyperbranched polyglycerol (hPG) functionalization in maintaining adsorption capacity, favorable kinetics, and structural stability during regeneration.

Overall, these results confirm the superior recyclability and operational stability of hPG-MOF compared with PVA-MOF, positioning hPG-MOF polymer composites as promising candidates for scalable dye removal in industrial wastewater treatment. Despite the encouraging adsorption performance, certain limitations should be acknowledged, including the reduced regeneration efficiency of PVA-MOF and the powdered nature of the materials, which may necessitate immobilization or shaping strategies for large-scale practical applications. Also, durability of materials can be a limitation that should be improved for application in aqueous media.

## Conclusions

In this study, polymer–MOF composites based on polyvinyl alcohol (PVA) and hyperbranched polyglycerol (hPG) were successfully synthesized and evaluated as efficient adsorbents for the removal of cationic and anionic dyes from aqueous solutions. Comprehensive characterization confirmed the successful functionalization and formation of amorphous iron-based MOF structures. Among the two materials, hPG-MOF exhibited superior adsorption capacity, stability, and reusability over multiple adsorption–desorption cycles. Adsorption followed Langmuir isotherm behavior and pseudo-second-order kinetics, indicating monolayer chemisorption as the dominant mechanism. Thermodynamic analyses revealed an endothermic adsorption process, spontaneous for anionic dyes and temperature-dependent for cationic dyes. Overall, the results demonstrate the strong potential of hPG-MOF composites for sustainable wastewater treatment applications, while highlighting the importance of polymer architecture in adsorption performance and recyclability. However, limitations in material durability and reusability must be considered for large-scale applications.

## Supplementary Information

Below is the link to the electronic supplementary material.

Supplementary Material 1

## Data Availability

The datasets used and/or analysed during the current study available from the corresponding author on reasonable request.
