# Supplementary material for: Metal-organic frameworks with linear and branched polyol backbones for dye removal
Source: Sci Rep. 2026 May 28;16:16555. doi: 10.1038/s41598-026-37325-0 (PMC13216250; doi:10.1038/s41598-026-37325-0)
Supplement: Supplementary file 1 — Supplementary Material 1 [file 41598_2026_37325_MOESM1_ESM.docx]

**Supporting Information**

**Metal-Organic Frameworks with Linear and Branched Polyol Backbones for Dye Removal**

*Safoora Gazvineh, Mohsen Adeli*, Mohammad Nemati*

*Department of Organic Chemistry, Faculty of Chemistry, Lorestan University, Khorramabad 68141-54316, Iran*

**Corresponding author:* *adeli.*[*m@lu.ac.ir*](mailto:m@lu.ac.ir)*,* [*m.aadeli@fu-berlin.de*](mailto:m.aadeli@fu-berlin.de)*(M. Adeli)*

**Experimental…………………………………………………………….………….….…..…..3**

Synthesis of PVA-OMs………………….…...........................................……………...…....….3

Synthesis of PVA-AIP…….….............................................................................................…...3

Synthesis of PVA-MOF…………...……………………..…………………………………......3

Synthesis of hPG-OMs………............................................................………..……...…….…...3

Synthesis of hPG-AIP…………………………......……………………...………………….…3

Synthesis of hPG-MOF………………......................………………...…………………….…..3

Characterization............................................................................................................................4

Adsorption Experiments...............................................................................................................4

**Results…………………………………………………………………...……………..…….…4**

Table S1. Comparative elemental analysis of starting materials and synthesized MOF/polymer composites: (a) EDAX results and (b) CHNS analysis, presented for different stages of the synthesis process………………………………………………………………...….……..…….4

Figure S1. UV-Visible absorption spectra of (a) PVA and PVA-OMS, and (b) hPG and hPG-OMS..............................................................................................................................................5

Table S2. The BET surface area, pore volume, and average pore diameter of the synthesized

PVA-MOF and hPG-MOF…………………………………………………………..…….…....5

Table S3. Adsorption capacities (mg·g⁻¹) of the synthesized PVA-MOF and hPG-MOF for the removal of Rhodamine B (Rh B), Methylene Blue (MB), and Fluorescein (FL), in comparison with analogous systems reported in the literature……………………………………………….6

**Supplementary Kinetic Studies..................................................................................................7**

Figure S2. Kinetic models describing the adsorption mechanisms of MB, RhB, and FL dyes on PVA-MOF (a) and hPG-MOF (b) based on the pseudo-first-order kinetic model………..…….7

Pseudo-first-order kinetic model ………………………...………………………...……...…….7

Pseudo-second-order kinetic model ..............................................................................................8

Table S4. Kinetic parameters for the adsorption of various dyes onto PVA-MOF and hPG-MOF…………………………………………………………………………………………….8

**Supplementary Adsorption Isotherm Studies..........................................................................9**

Langmuir isotherm........................................................................................................................9

Freundlich isotherm .....................................................................................................................10

Figure S3. (a) Langmuir isotherm plots for the adsorption of MB, Rh B, and FL onto PVA-MOF and hPG-MOF. (b) Freundlich isotherm plots describing the adsorption behavior of MB, Rh B, and FL on the same MOFs. (c) Experimental adsorption isotherms of MB, Rh B, and FL at various equilibrium concentrations……………………………………..………….……………10

Table S5. Isotherm parameters for Langmuir and Freundlich models describing the adsorption of MB, RhB, and FL dyes onto PVA-MOF and hPG-MOF……………………....………………11

Table S6. RL values for the adsorption of varying concentrations of MB, RhB, and FL dyes by PVA-MOF and hPG-MOF……………………………………………………….………….…..12

**Supplementary Thermodynamic Studies…………….......................……………..………….13**

Table S7. Various parameters including removal efficiency, adsorption capacity, and adsorption equilibrium constant for the adsorption of MB, RhB, and FL dyes by PVA-MOF and hPG-MOF at 25 °C………………………………….……………………….……………..………………..14

Figure S4. Plots of ln (K_ad_) versus 1/T for the adsorption of MB (a), RhB (b), and FL (c) dyes on PVA-MOF and hPG-MOF at various temperatures……………………..…………….………...15

Table S8. Thermodynamic parameters for the adsorption of MB, RhB, and FL dyes by PVA-MOF and hPG-MOF……………………………………………………..……………….……..15

**Supplementary Reusability Data**...............................................................................................16

Table S9. Adsorption capacity and adsorption equilibrium constant parameters for MB, RhB, and FL dyes by PVA-MOF (a) and hPG-MOF (b) after 24 hours of shaking at 25 °C with dye solutions (5 mL,50 ppm)……………………………………………………….………………..16

**References………………………………...………………………………….…………………17**

# Experimental

*Synthesis of PVA-OMs*

PVA (0.2 g, 0.0028 mmol) was dissolved in DMF (10 mL) at 110 °C. After cooling, Et₃N (1 mL) was added and stirred 2 h. MsCl (1.78 mL, 23 mmol) was added dropwise in an ice bath over 1 h, then stirred at room temperature for 3 days. The product was crystallized from acetonitrile (yield: 69.7%).

*Synthesis of PVA-AIP*

PVA-OMs (0.1 g, 0.000736 mmol) was dissolved in acetonitrile (25 mL) with K₂CO₃ (0.1 g, 0.724 mmol) and AIP (0.11 g, 0.6 mmol). Reflux at 80 °C for 24 h. Purification via dialysis in DMF, D₂O, and EtOH. Yield: 84.04%.

*Synthesis of PVA-MOF*

PVA-AIP and FeCl₃·6H₂O mixed in 2:1 ratio in DMF (25 mL). Stirred 2 h, then autoclaved at 110 °C for 20 h. Washed with DMF and EtOH, dried in vacuum oven at 50 °C. Yield: 70.39%.

*Synthesis of hPG-OMs*

hPG (0.258 g, 0.0516 mmol) dissolved in DMF (10 mL) under N₂. Et₃N (1 mL) added at 0 °C. MsCl (1.74 mL, 22.47 mmol) added dropwise, then refluxed at room temperature 24 h. Purification by dialysis in acetonitrile. Yield: 78.66%.

*Synthesis of hPG-AIP*

hPG-OMs (0.1 g, 0.0156 mmol) dissolved in acetonitrile (25 mL) with K₂CO₃ (0.1 g, 0.724 mmol) and AIP (0.234 g, 1.29 mmol). Stirred at 80 °C for 24 h. Dialysis purification (DMF, D₂O, EtOH), dried at 50 °C. Yield: 83.66%.

*Synthesis of hPG-MOF*

hPG-AIP and FeCl₃·6H₂O reacted as for PVA-MOF. Purified and dried similarly.

*Characterization*

- FTIR, XRD, SEM/EDX, BET, Zeta, UV–Vis DRS, NMR: as in main manuscript.
- CHNS analysis: Table S1, confirms elemental composition consistent with EDX.

*Adsorption experiments*

As in main manuscript, with detailed dye concentrations, volumes, incubation times, and calculation formulas. Quantitative adsorption data and removal efficiencies in Tables S4–S9.

# Results

**Table S1**. Comparative elemental analysis of starting materials and synthesized MOF/polymer composites: (a) EDAX results and (b) CHNS analysis, presented for different stages of the synthesis process.

a

| **Element** | **%A**  **%W** | **%C** | **%O** | **%S** | **%N** | **%Fe** |
| --- | --- | --- | --- | --- | --- | --- |
| **PVA** | | 65.42  58.68 | 34.58  41.32 | -  - | -  - | -  - |
| **PVA-OMs** | | 65.93  55.37 | 28.81  32.23 | 5.26  12.40 | -  - | -  - |
| **PVA-AIP** | | 58.09  51.85 | 30.55  36.32 | -  - | 11.36  11.83 | -  - |
| **PVA-MOF** | | 57.95  47.20 | 27.09  35.64 | -  - | 11.50  11.39 | 3.46  5.77 |
| **hPG** | | 73.55  67.61 | 26.45  32.39 | -  - | -  - | -  - |
| **hPG-OMs** | | 62.51  52.44 | 32.91  36.78 | 4.58  10.78 | -  - | -  - |
| **hPG-AIP** | | 48.22  41.70 | 42.41  48.85 | -  - | 9.37  9.45 | -  - |
| **hPG-MOF** | | 57.66  50.11 | 34.06  39.43 | -  - | 7.35  7.45 | 0.93  3.01 |

b

| **Element** | **%C** | **%H** | **%S** | **%N** |
| --- | --- | --- | --- | --- |
| **PVA** | 53.48 | 8.71 | - | - |
| **PVA-OMs** | 40.85 | 5.72 | 10.36 | - |
| **PVA-AIP** | 42.62 | 5.49 | 0.92 | 5.69 |
| **PVA-MOF** | 49.26 | 6.21 | 0.38 | 5.53 |
| **hPG** | 50.28 | 11.71 | - | - |
| **hPG-OMs** | 38.01 | 6.42 | 11.71 | - |
| **hPG-AIP** | 42.81 | 6.07 | 0.86 | 5.67 |
| **hPG-MOF** | 46.52 | 6.18 | 0.48 | 5.30 |


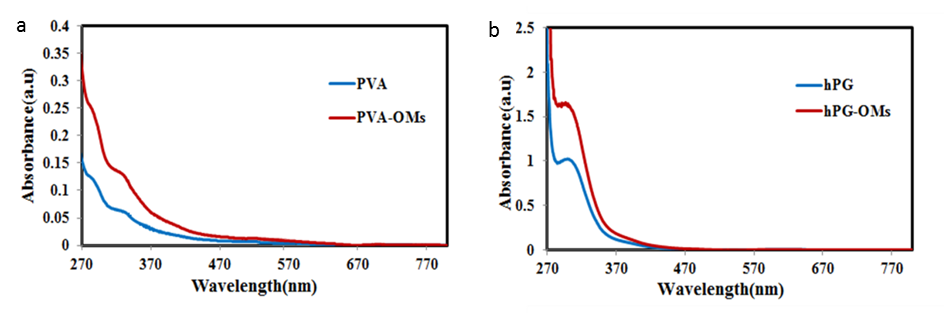


**Figure S1.** UV-Visible absorption spectra of (a) PVA and PVA-OMs, and (b) hPG and hPG-OMs.

**Table S2**. The BET surface area, pore volume, and average pore diameter of the synthesized

PVA-MOF and hPG-MOF.

| **Compound** | **BET Surface Area** | **BJH Adsorption cumulative volume of pores** | **Average pore diameter (4V/A by BET)** |
| --- | --- | --- | --- |
| **PVA-MOF** | 15.509 m².g^-1^ | 0.048105 cm³.g^-1^ | 11.8 nm |
| **hPG-MOF** | 18.609 m².g^-1^ | 0.051623 cm³.g^-1^ | 11.12 nm |

**Table S3.** Adsorption capacities (mg·g⁻¹) of the synthesized PVA-MOF and hPG-MOF for the removal of Rhodamine B (Rh B), Methylene Blue (MB), and Fluorescein (FL), in comparison with analogous systems reported in the literature.

| **Adsorbent** | **Rhodamine B (mg.g^-1^)** | **Methylene Blue (mg.g^-1^)** | **Fluorescein (mg.g^-1^)** | **Reference** |
| --- | --- | --- | --- | --- |
| **PVA-MOF (This Work)** | 128.17 | 128.24 | 124.77 | This study |
| **hPG-MOF (This Work)** | 131.46 | 135.34 | 128.31 | This study |
| **PNIPAM-Sponge** | 245 | 98 | 231 | 1 |
| **UiO-66-NH2** | 104.20 | 106.80 | 95.10 | 2 |
| **ZIF‑8** | 2500 | 40 | - | 3 |
| **silica microspheres (SM) with controlled hydrophobicity** | 55-146 | - | 26-132 | 4 |
| **MOF-199/CCF** | 659.6 | - | - | 5 |
| **Zn‑MOF** | 55.34 | - | - | 6 |
| **UiO-66-NO2** | 41.7 | - | - | 7 |
| **MOF-5/PANI** | 135 | - | - | 8 |
| **MIL‑100(Fe)** | 763 | - | - | 9 |
| **MOF‑235** | 252 | - | - | 10 |
| **Tweezers-Like­­Adsorbent (CS-Ac-An)** | - | - | 61.8 | 11 |
| **(MV)[BiI3Cl2]** | - | - | 793 | 12 |

**Supplementary Kinetic Studies**

To further elucidate the adsorption kinetics of Methylene Blue (MB), Rhodamine B (RhB), and Fluorescein (FL) onto PVA-MOF and hPG-MOF, the experimental data were analyzed using pseudo-first-order and pseudo-second-order kinetic models. The detailed fitting procedures, linearized plots, and calculated kinetic parameters are presented here to support the conclusions discussed in the main manuscript.


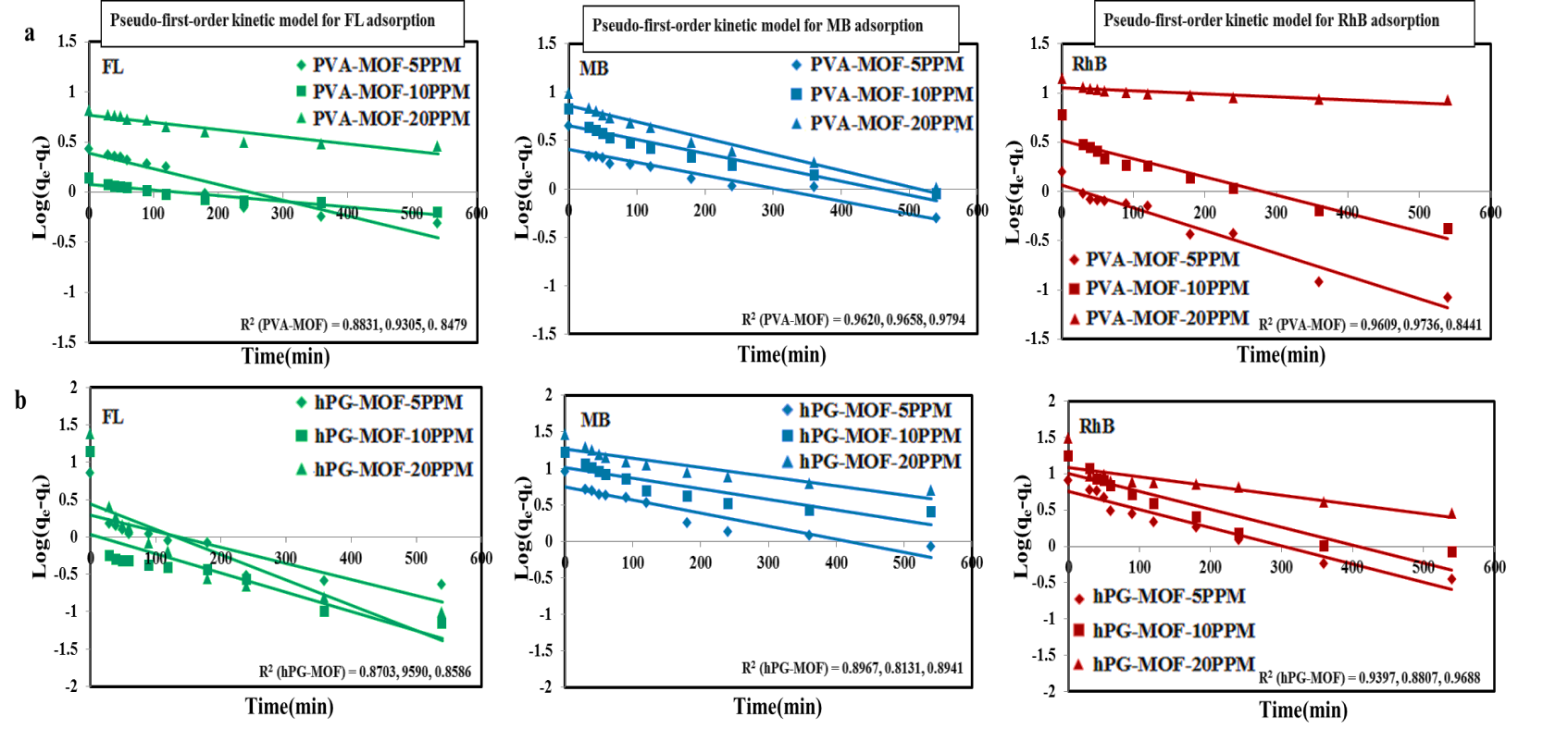


**Figure S2.** Kinetic models describing the adsorption mechanisms of MB, RhB, and FL dyes on PVA-MOF (a) and hPG-MOF (b) based on the pseudo-first-order kinetic model. Data fitting indicates that physisorption predominantly governs the dye adsorption process. All experiments were conducted under shaking conditions to evaluate the adsorption kinetics and the dye uptake capacity of MOFs for different dyes.

**Pseudo-first-order kinetic model**

The pseudo-first-order kinetic model is expressed by the following linear equation:

ln(q_e_ - q_t_) = ln qe – k_1_t (1)

where qₑ (mg.g⁻¹) and q_t_ (mg.g⁻¹) are the adsorption capacities at equilibrium and at time t (min), respectively, and k₁ (min⁻¹) is the pseudo-first-order rate constant. Linear plots of ln(qₑ -q_t_) versus time were constructed for each dye at different initial concentrations (Figure S2 and Table S4).

**Pseudo-second-order kinetic model**

The pseudo-second-order kinetic model is described by the following equation:

t/q_t_ = 1/(k_2_q_e_^2^) + t/q_e_  (2)

where k₂ (g.mg⁻¹.min⁻¹) is the pseudo-second-order rate constant. The kinetic parameters were obtained from the linear plots of t/q_t_ versus time for all dyes (Table S4).

**Table S4.** Kinetic parameters for the adsorption of various dyes onto PVA-MOF and hPG-MOF. The table summarizes the calculated values based on kinetic models, providing insights into the adsorption rates and mechanisms of dye uptake by both polymer-metal-organic frameworks.

| **Adsorbent** | **C(mg.L^-1^)** | **First order kenetic** | | | **Second order kenetic** | | |
| --- | --- | --- | --- | --- | --- | --- | --- |
|  |  | K_1_ | q_e_ | R^2^ | K_2_ | q_e_ | R^2^ |
| PVA-MOF **MB** | 5 | 0.0028 | 0.4380 | 0.962 | 0.0062 | 4.0519 | 0.9907 |
|  | 10 | 0.0030 | 0.2438 | 0.9658 | 0.0027 | 6.2854 | 0.9977 |
|  | 20 | 0.0037 | 0.1475 | 0.9794 | 0.0013 | 9.8912 | 0.9972 |
| PVA-MOF **Rh B** | 5 | 0.0051 | 0.9221 | 0.9609 | 0.0084 | 1.6790 | 0.9846 |
|  | 10 | 0.0039 | 0.3426 | 0.9736 | 0.0044 | 5.8720 | 0.9977 |
|  | 20 | 0.005 | 0.0917 | 0.8441 | 0.0038 | 6.0680 | 0.9988 |
| PVA-MOF **FL** | 5 | 0.0035 | 0.4160 | 0.8831 | 0.0009 | 3.6630 | 0.8905 |
|  | 10 | 0.0012 | 0.8632 | 0.9305 | 0.0100 | 0.8587 | 0.9816 |
|  | 20 | 0.0016 | 0.1742 | 0.8479 | 0.0007 | 5.6022 | 0.9247 |

| **Adsorbent** | **C(mg.L^-1^)** | **First order kenetic** | | | **Second order kenetic** | | |
| --- | --- | --- | --- | --- | --- | --- | --- |
|  |  | K_1_ | q_e_ | R^2^ | K_2_ | q_e_ | R^2^ |
| hPG-MOF **MB** | 5 | 0.0039 | 0.1990 | 0.8967 | 0.0027 | 8.9206 | 0.9958 |
|  | 10 | 0.0030 | 0.1073 | 0.8131 | 0.0013 | 15.7978 | 0.998 |
|  | 20 | 0.0025 | 0.0596 | 0.8941 | 0.0008 | 26.3852 | 0.9996 |
| hPG-MOF **Rh B** | 5 | 0.0055 | 0.1884 | 0.9397 | 0.0014 | 9.0416 | 0.9849 |
|  | 10 | 0.0053 | 0.1123 | 0.8807 | 0.0012 | 18.5874 | 0.9973 |
|  | 20 | 0.0023 | 0.0996 | 0.9688 | 0.0007 | 30.3951 | 0.9995 |
| hPG-MOF **FL** | 5 | 0.0042 | 0.6831 | 0.8703 | 0.0124 | 6.9881 | 0.9993 |
|  | 10 | 0.0041 | 1.5911 | 0.959 | 0.0308 | 13.6799 | 1 |
|  | 20 | 0.0062 | 0.5855 | 0.8586 | 0.0123 | 24.2718 | 1 |

**Supplementary Adsorption Isotherm Studies**

Adsorption isotherm analyses were conducted to evaluate the equilibrium interaction between dye molecules and the synthesized adsorbents. The experimental data were fitted using Langmuir and Freundlich isotherm models, and the detailed fitting results are presented below.

**Langmuir isotherm**

The Langmuir isotherm model is represented by the following linear equation:

C_e_/q_e_ = 1/K_l_q_m_+ C_e_/q_m_ (3)

where q_m_ (mg.g⁻¹) is the maximum adsorption capacity and K_L_ (L.mg⁻¹) is the Langmuir constant related to adsorption affinity.

**Freundlich isotherm**

The Freundlich isotherm model is expressed as:

ln q_e_ = ln K_f_ + 1/n lnC_e_ (4)

where K_F_ is the Freundlich constant and 1/n represents surface heterogeneity ( Figure S3 and Table S5,S6).


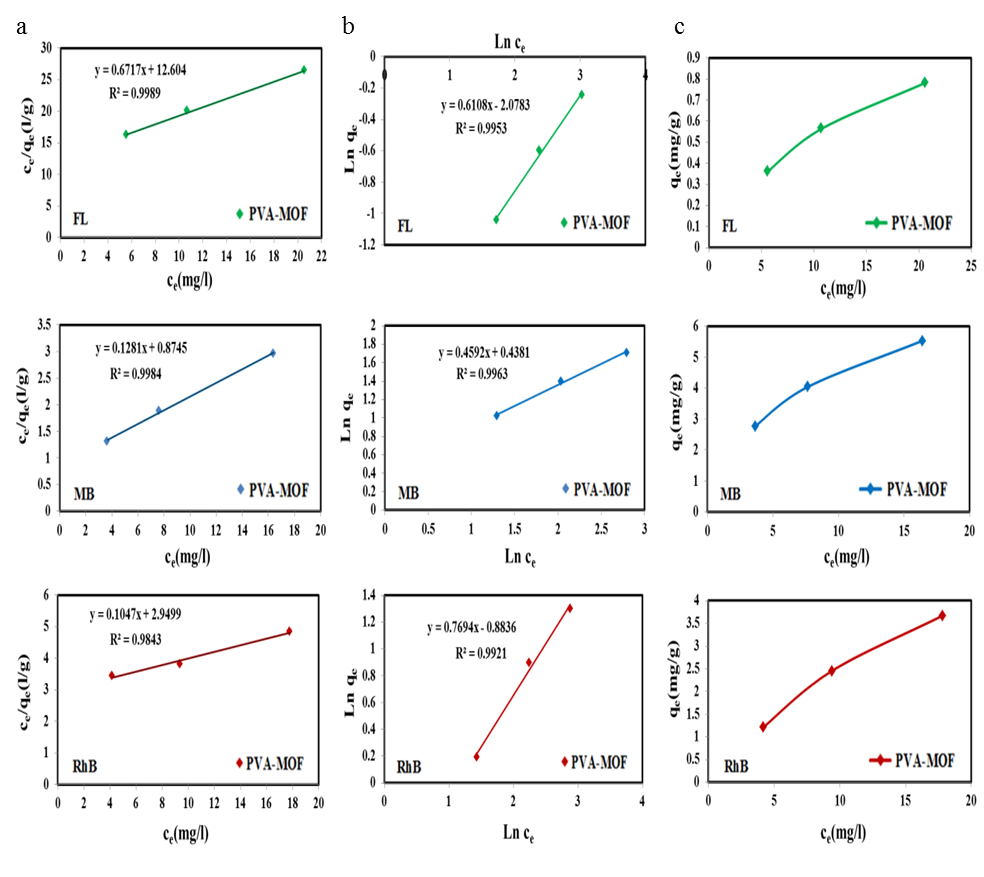


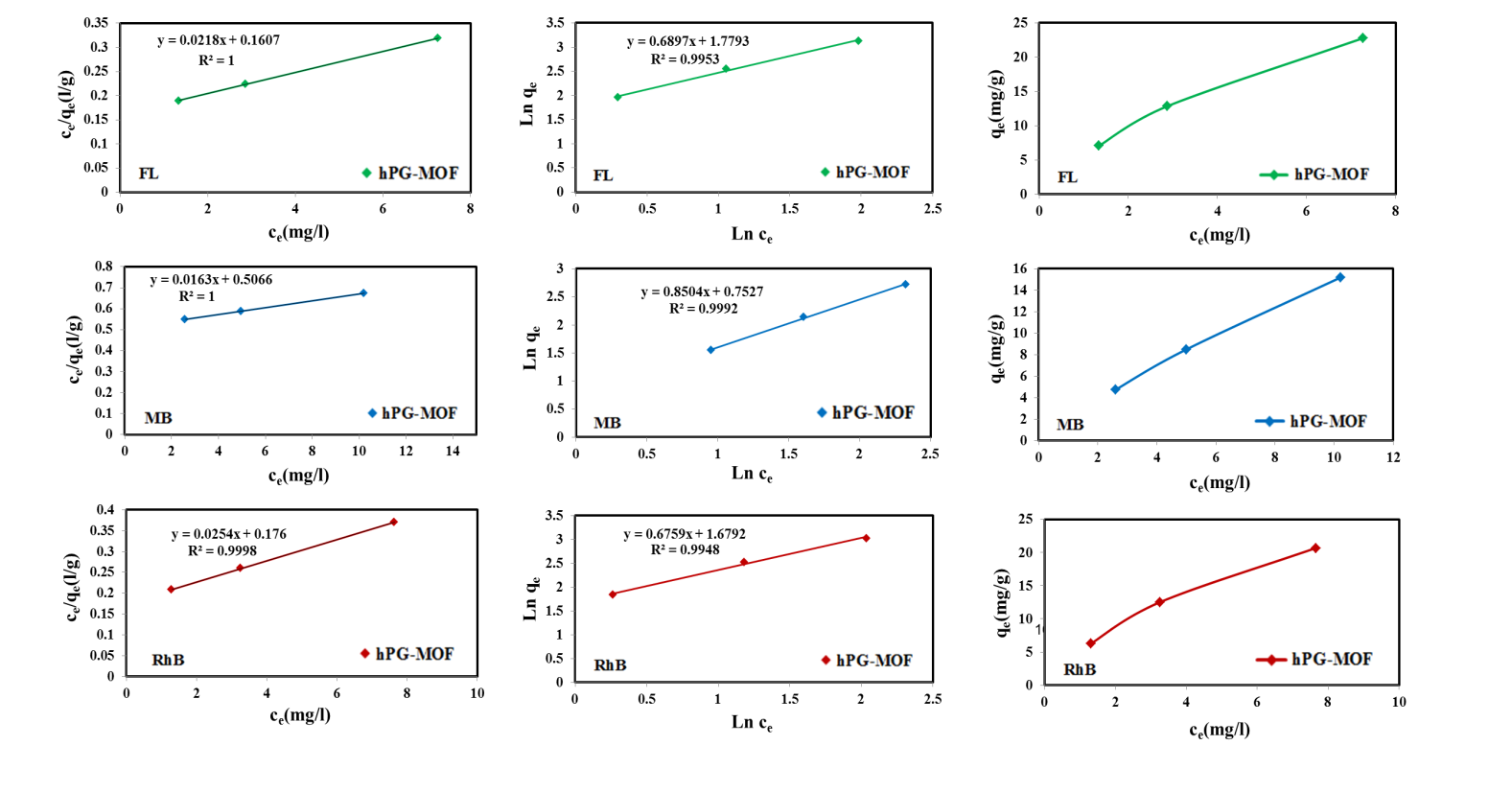


**Figure S3.** (a) Langmuir isotherm plots for the adsorption of MB, Rh B, and FL onto PVA-MOF and hPG-MOF. (b) Freundlich isotherm plots describing the adsorption behavior of MB, Rh B, and FL on the same MOFs. (c) Experimental adsorption isotherms of MB, Rh B, and FL at various equilibrium concentrations. All adsorption experiments were performed under continuous shaking to evaluate the affinity and adsorption capacity of the composites for each dye.

**Table S5.** Isotherm parameters for Langmuir and Freundlich models describing the adsorption of MB, RhB, and FL dyes onto PVA-MOF and hPG-MOF. This table presents the equilibrium constants and adsorption capacities, providing detailed insight into the adsorption behavior and affinity of MOFs toward different dyes.

| **Adsorbent** | **Langmuir Isotherm** | | | **Freundlich Isotherm** | | | |
| --- | --- | --- | --- | --- | --- | --- | --- |
|  | q_max_ | k_l_ | R^2^ | k_f_ | 1/n | n | R^2^ |
| PVA-MOF **MB** | 7.8064 | 0.1465 | 0.9984 | 1.5498 | 0.4592 | 2.1777 | 0.9963 |
| PVA-MOF **RhB** | 5.4318 | 0.0898 | 0.9989 | 0.6762 | 0.5615 | 1.2997 | 0.9960 |
| PVA-MOF **FL** | 1.4888 | 0.0533 | 0.9989 | 7.9909 | 0.6108 | 1.6385 | 0.9953 |

| **Adsorbent** | **Langmuir Isotherm** | | | **Freundlich Isotherm** | | | |
| --- | --- | --- | --- | --- | --- | --- | --- |
|  | q_max_ | k_l_ | R^2^ | k_f_ | 1/n | n | R^2^ |
| hPG-MOF **MB** | 61.3497 | 0.0322 | 1 | 2.1227 | 0.8504 | 1.1760 | 0.9992 |
| hPG-MOF **RhB** | 39.3701 | 0.1443 | 0.9998 | 5.3613 | 0.6759 | 1.4795 | 0.9948 |
| hPG-MOF **FL** | 45.8716 | 0.1357 | 1 | 5.9257 | 0.6897 | 1.4499 | 0.9953 |

**Table S6.** RL values for the adsorption of varying concentrations of MB, RhB, and FL dyes by PVA-MOF and hPG-MOF. The RL parameter evaluates the favorability of the adsorption process, reflecting the interaction affinity between dyes and MOFs at different initial dye concentrations.

| **Adsorbent** | **C(mg.L^-1^)** | **R_L_** |
| --- | --- | --- |
| PVA-MOF  **MB** | 5 | 0.5772 |
|  | 10 | 0.4057 |
|  | 20 | 0.2545 |
| PVA-MOF  **RhB** | 5 | 0.6901 |
|  | 10 | 0.5269 |
|  | 20 | 0.3577 |
| PVA-MOF  **FL** | 5 | 0.7896 |
|  | 10 | 0.6523 |
|  | 20 | 0.4840 |

| **Adsorbent** | **C(mg.L^-1^)** | **R_L_** |
| --- | --- | --- |
| hPG-MOF  **MB** | 5 | 0.8613 |
|  | 10 | 0.7564 |
|  | 20 | 0.6083 |
| hPG-MOF  **RhB** | 5 | 0.5809 |
|  | 10 | 0.4093 |
|  | 20 | 0.2573 |
| hPG-MOF  **FL** | 5 | 0.5958 |
|  | 10 | 0.4243 |
|  | 20 | 0.2693 |

**Supplementary Thermodynamic Studies**

Thermodynamic analyses were performed to evaluate the feasibility and energetic nature of the adsorption process. The thermodynamic parameters including Gibbs free energy change (ΔG°), enthalpy change (ΔH°), and entropy change (ΔS°) were calculated using equilibrium adsorption data obtained at different temperatures ( Figure S4 and Table S7,S8).

Thermodynamic equations

The adsorption equilibrium constant (K_ad_) was calculated as:

K_ad_=C_0_-C_e_/C_e_  (5)

The relationship between K_ad_ and temperature is given by:

(6) Ln k_ad_=ΔS/R-ΔH/RT

The Gibbs free energy change was calculated using:

ΔGᵒ=ΔHᵒ-TΔSᵒ (7)

**Table S7.** Various parameters including removal efficiency, adsorption capacity, and adsorption equilibrium constant for the adsorption of MB, RhB, and FL dyes by PVA-MOF and hPG-MOF at 25 °C. This table summarizes the performance metrics of MOFs under controlled temperature conditions.

| **q_t_(mg.g^-1^)** | **%R** | **Ln k_ad_** | **k_ad_** | **T(ᵒk)** | **Adsorbent** |
| --- | --- | --- | --- | --- | --- |
| 0.7565 | 4.5272 | -3.0487 | 0.0474 | 323 | PVA-MOF **MB** |
| 1.0737 | 6.4257 | -2.6785 | 0.0687 | 333 |  |
| 1.7814 | 10.6608 | -2.1259 | 0.1193 | 343 |  |
| 1.9300 | 10.7513 | -2.1164 | 0.1205 | 323 | PVA-MOF **Rh B** |
| 2.3231 | 12.9413 | -1.9061 | 0.1487 | 333 |  |
| 3.0141 | 16.7906 | -1.6005 | 0.2018 | 343 |  |
| 0.0742 | 0.4083 | -5.4968 | 0.0041 | 323 | PVA-MOF  **FL** |
| 9.2428 | 50.8371 | 0.0335 | 1.0341 | 333 |  |
| 14.5137 | 79.8285 | 1.3756 | 3.9575 | 343 |  |

| **q_t_(mg.g^=1^)** | **%R** | **Ln k_ad_** | **k_ad_** | **T(ᵒk)** | **Adsorbent** |
| --- | --- | --- | --- | --- | --- |
| 1.2079 | 7.1506 | -2.5638 | 0.0770 | 323 | hPGMOF  **MB** |
| 3.1479 | 18.6349 | -1.4739 | 0.2290 | 333 |  |
| 4.3680 | 25.8577 | -1.0534 | 0.3488 | 343 |  |
| 0.6433 | 3.6590 | -3.2707 | 0.0380 | 323 | hPG-MOF  **Rh B** |
| 2.0372 | 11.5869 | -2.0321 | 0.1311 | 333 |  |
| 5.4920 | 31.2373 | -0.7891 | 0.4543 | 343 |  |
| 12.8805 | 73.5481 | 1.0226 | 2.7804 | 323 | hPG-MOF  **FL** |
| 14.8478 | 84.7817 | 1.7176 | 5.5710 | 333 |  |
| 15.5902 | 89.0208 | 2.0929 | 8.1081 | 343 |  |


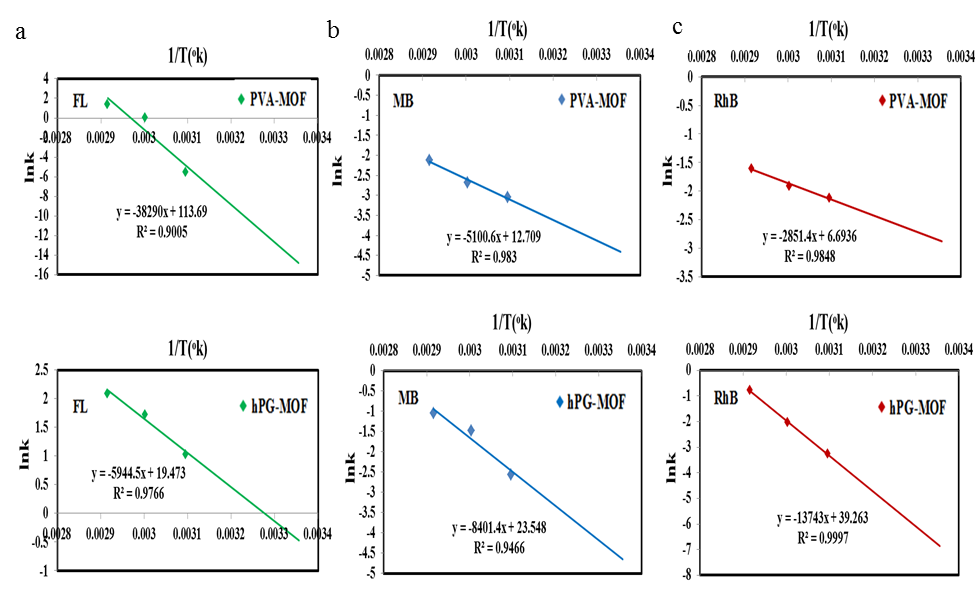


**Figure S4.** Plots of ln (K_ad_) versus 1/T for the adsorption of MB (a), RhB (b), and FL (c) dyes on PVA-MOF and hPG-MOF at various temperatures. These graphs illustrate the temperature dependence of the adsorption equilibrium constant, providing insight into the thermodynamic behavior and adsorption affinity of MOFs.

**Table S8.** Thermodynamic parameters for the adsorption of MB, RhB, and FL dyes by PVA-MOF and hPG-MOF. The table includes values of enthalpy, entropy, and Gibbs free energy changes, providing insights into the spontaneity and nature of the adsorption processes.

| **R^2^** | **ΔGᵒ(kj.mol^-1^)** | **T(ᵒk)** | **ΔSᵒ(j.mol^-1^.k^-1^)** | **ΔHᵒ(kj.mol^-1^)** | **C(mg.L^-1^)** | **Adsorbent** |
| --- | --- | --- | --- | --- | --- | --- |
| 0.9830 | +10.9078 | 298 | +0.1057 | +42.4064 | 10 | PVA-MOF **MB** |
|  | +8.2653 | 323 |  |  |  |  |
|  | +7.2083 | 333 |  |  |  |  |
|  | +6.1513 | 343 |  |  |  |  |
| 0.9848 | +7.1079 | 298 | +0.0557 | +23.7065 | 10 | PVA-MOF **Rh B** |
|  | +5.7154 | 323 |  |  |  |  |
|  | +5.1584 | 333 |  |  |  |  |
|  | +4.6014 | 343 |  |  |  |  |
| 0.9005 | +36.6735 | 298 | +0.9452 | +318.3431 | 10 | PVA-MOF **FL** |
|  | +13.0435 | 323 |  |  |  |  |
|  | +3.5915 | 333 |  |  |  |  |
|  | -5.8605 | 343 |  |  |  |  |

| **R^2^** | **ΔGᵒ(kj.mol^-1^)** | **T(ᵒk)** | **ΔSᵒ(j.mol^-1^.k^-1^)** | **ΔHᵒ(kj.mol^-1^)** | **C(mg.L^-1^)** | **Adsorbent** |
| --- | --- | --- | --- | --- | --- | --- |
| 0.9466 | +11.5008 | 298 | +0.1958 | +69.8492 | 10 | hPG-MOF **MB** |
|  | +6.6058 | 323 |  |  |  |  |
|  | +4.6478 | 333 |  |  |  |  |
|  | +2.6898 | 343 |  |  |  |  |
| 0.9997 | +16.9921 | 298 | +0.3264 | +114.2593 | 10 | hPG-MOF **Rh B** |
|  | +8.8321 | 323 |  |  |  |  |
|  | +5.5681 | 333 |  |  |  |  |
|  | +2.3041 | 343 |  |  |  |  |
| 0.9766 | +1.1764 | 298 | +0.1619 | +49.4226 | 10 | hPG-MOF **FL** |
|  | -2.8711 | 323 |  |  |  |  |
|  | -4.4901 | 333 |  |  |  |  |
|  | -6.1091 | 343 |  |  |  |  |

**Supplementary Reusability Data**

The adsorption capacity and adsorption equilibrium constants of MB, RhB, and FL dyes by PVA-MOF and hPG-MOF after adsorption–desorption cycles are summarized in Table S9 to support the recyclability discussion presented in the main manuscript.

**Table S9.** Adsorption capacity and adsorption equilibrium constant parameters for MB, RhB, and FL dyes by PVA-MOF (a) and PG-MOF (b) after 24 hours of shaking at 25 °C with dye solutions (5 mL, 50 ppm). This table summarizes the equilibrium adsorption performance of MOFs under controlled conditions.

a

| **Absorbent** | **Dyes (50 ppm)** | **Maximum dye adsorption (mg.g^-1^)** |
| --- | --- | --- |
| **H_2_O-Gordab Sang khorramabad** | RhB | 126.31 |
|  | MB | 118.17 |
|  | FL | 105.59 |
| **H_2_O -Khorram Rud khorramabad** | RhB | 125.63 |
|  | MB | 120.08 |
|  | FL | 110.16 |
| **H_2_O- Drinking water Khorramabad** | RhB | 127.78 |
|  | MB | 120.21 |
|  | FL | 121.74 |
| **Deionized water** | RhB | 128.17 |
|  | MB | 128.24 |
|  | FL | 124.77 |

b

| **Absorbent** | **Dyes (50 ppm)** | **Maximum dye adsorption (mg.g^-1^)** |
| --- | --- | --- |
| **H_2_O-Gordab Sang khorramabad** | MB | 129.01 |
|  | RhB | 128.67 |
|  | FL | 119.40 |
| **H_2_O -Khorram Rud khorramabad** | MB | 132.01 |
|  | RhB | 128.88 |
|  | FL | 123.85 |
| **H_2_O- Drinking water Khorramabad** | MB | 134.64 |
|  | RhB | 131.14 |
|  | FL | 125.19 |
| **Deionized water** | MB | 135.34 |
|  | RhB | 131.46 |
|  | FL | 128.31 |

**References**

1. Gazvineh, S.; Beyranvand, S.; Saki, S.; Nemati, M.; Ludwig, K.; Cheng, C.; Amsalem, P.; Schultz, T.; Adeli, M., Thermoresponsive Scaffolds Fabricated Using Covalent Organic Frameworks for the Selective Removal of Water Contaminants. *Materials Advances* **2024**.

2. Fang, M.-D.; Drobek, M.; Cot, D.; Montoro, C.; Semsarilar, M., A Straightforward Method to Prepare MOF-Based Membranes via Direct Seeding of MOF-Polymer Hybrid Nanoparticles. *Membranes* **2023,** *13*.

3. Zhao, J.; Li, X.; Wang, Y.; Chen, L. Efficient removal of dyes by ErIII-based metal-organic frameworks. *Journal of Molecular Structure* **2024**, 138345.

4. Melnyk, I. V.; Tomina, V. V.; Stolyarchuk, N. V.; Seisenbaeva, G. A.; Kessler, V. G., Organic dyes (acid red, fluorescein, methylene blue) and copper(II) adsorption on amino silica spherical particles with tailored surface hydrophobicity and porosity. *Journal of Molecular Liquids* **2021,** *336*, 116301.

5. Liang, Z.; Liang, Y.; Yu, P.; Wang, X., Ultrasonic-assisted in situ synthesis of MOF-199 on the surface of carboxylated cellulose fibers for efficient adsorption of methylene blue. *RSC Advances* **2024,** *14*, 15095 - 15105.

6. Elsherbiny, A. S.; Rady, A.; Abdelhameed, R. M.; Gemeay, A. H., Efficiency and selectivity of cost-effective Zn-MOF for dye removal, kinetic and thermodynamic approach. *Environmental Science and Pollution Research International* **2023,** *30*, 106860 - 106875.

7. Dinh, H. T.; Tran, N. T.; Trinh, D. X., Investigation into the Adsorption of Methylene Blue and Methyl Orange by UiO-66-NO2 Nanoparticles. *Journal of Analytical Methods in Chemistry* **2021,** *2021*.

8. Jevremović, A.; Savić, M.; Janošević Ležaić, A.; Krstić, J. B.; Gavrilov, N.; Bajuk-Bogdanović, D. V.; Milojević-Rakić, M.; Ćirić‐Marjanović, G., Environmental Potential of Carbonized MOF-5/PANI Composites for Pesticide, Dye, and Metal Cations—Can They Actually Retain Them All? *Polymers* **2023,** *15*.

9. Tan, Z., GO-MOF for organic dye adsorption. *RSC Advances* **2022,** *12* (34), 21411-21420.

10. Li, X., UiO-66-NH2 MOFs for water purification. *Materials Today Chemistry* **2022,** *23*, 100624.

11. B. Vafakish and L. D. Wilson, *Surfaces* **2019**, 2, 468-484.

12. J. Wang, J. Zheng, S. Wang, J. Sun, Y. Guo, H. Wang, S. Huang, Y. Li and C. Wang,

*Materials Letters* **2019**, 254, 419-422.
